# Phosphate–phosphate oligomerization drives higher order co-assemblies with stacks of cyanostar macrocycles†
†Electronic supplementary information (ESI) available: Experimental procedures, ^1^H NMR titrations, ^1^H NMR variable temperature experiments, ^1^H NMR diffusion studies, ESI-MS experiments, and X-ray crystallographic data. CCDC 1588590 and 1588591. For ESI and crystallographic data in CIF or other electronic format see DOI: 10.1039/c7sc05290a


**DOI:** 10.1039/c7sc05290a

**Published:** 2018-02-20

**Authors:** Elisabeth M. Fatila, Maren Pink, Eric B. Twum, Jonathan A. Karty, Amar H. Flood

**Affiliations:** a Department of Chemistry , Indiana University , Bloomington , IN 47405 , USA . Email: aflood@indiana.edu

## Abstract

The importance of phosphate in biology and chemistry has long motivated investigation of its recognition. Despite this interest, phosphate's facile oligomerization is only now being examined following the discovery of complexes of anion–anion dimers of hydroxyanions. Here we address how oligomerization dictates phosphate's recognition properties when engaged with planar cyanostar macrocycles that can also oligomerize by stacking. The crystal structure of cyanostar with phosphate shows an unprecedented tetrameric stack of cyanostar macrocycles threaded by a phosphate trimer, [H_2_PO_4_···H_2_PO_4_···H_2_PO_4_]^3–^. The solution behaviour, studied as a function of solvent quality, highlights how dimers and trimers of phosphate drive formation of higher order stacks of cyanostar into dimer, trimer and tetramer co-assemblies. Solution behaviors differ significantly from simpler complexes of bisulfate hydroxyanion dimers. Phosphate oligomerization is: (1) preferred over ion pairing with tetrabutylammonium cations, (2) inhibits disassembly of the complexes upon dilution, and (3) resists interference from competitive anion solvation. The phosphate oligomers also appear critical for stability; complexation of just one phosphate with cyanostars is unfavored. The cyanostar's ability to self-assemble is found to create a tubular, highly electropositive cavity that complements the size and shape of the phosphate oligomers as well as their higher charge. When given the opportunity, phosphate will cooperate with the receptor to form co-assembled architectures.

## Abstract 2

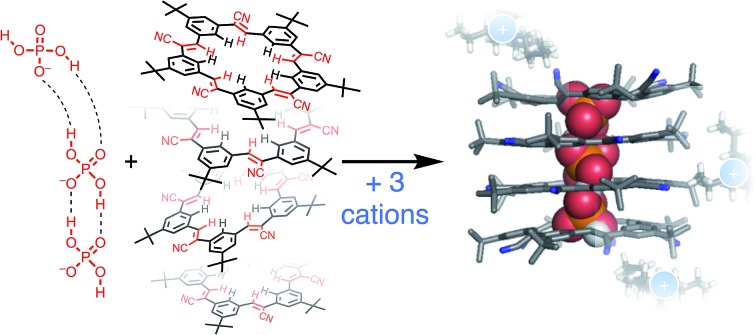
Phosphate–phosphate oligomerization is shown to drive its recognition properties as revealed in co-assemblies with stackable macrocyclic hosts.

## Introduction

**Fig. 1:**
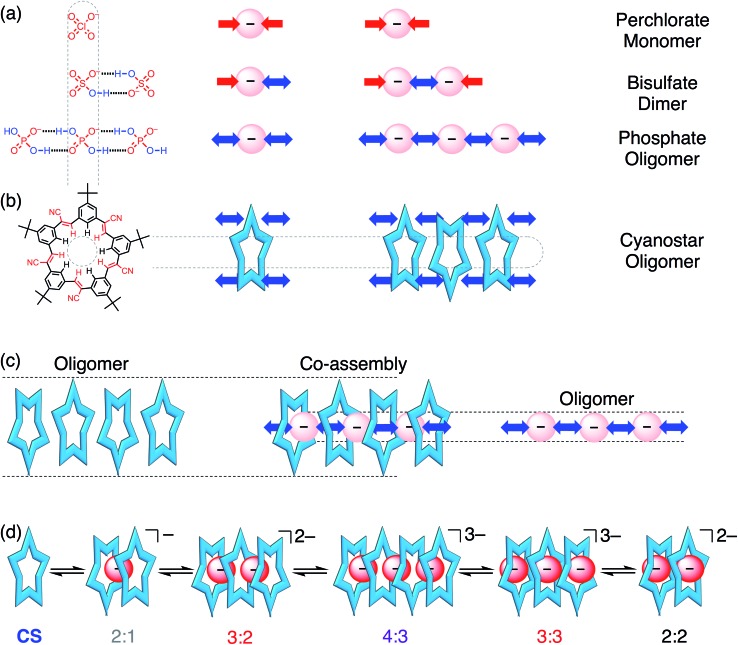
(a) Anions can self-associate by hydrogen bonding to different degrees; perchlorate (ClO_4_^–^) can only accept hydrogen bonds, bisulfate (HSO_4_^–^) can also donate one hydrogen bond, and phosphate (H_2_PO_4_^–^) is capable of donating two hydrogen bonds in a divergent manner. (b) Cyanostar macrocycles can capture size-matched anions and can undergo self-association. (c) The self-association of macrocycles and anions can produce co-assemblies of both. (d) Sequence of main equilibria and species present in organic solution during phosphate titration.

Phosphate is important to structural biology,^1^ an essential nutrient for ecosystems,^2^,^3^ and as a fertilizer it can produce eutrophication that harms natural waterways.^4^,^5^ These wide impact areas motivate studies to understand the supramolecular chemistry of phosphate and to contend with its complexities (*e.g.*, pH dependence,^6^–^8^ hydration energy). Perhaps the least examined complexity is phosphate's tendency to oligomerize (Fig. 1a) leading to names like “supra-anion”^7^ or “anion clusters”.^8^ As such, these species represent a new motif capable of supporting self-assembly.^9^,^10^ While commonplace in the solid state,^6^–^8^,^11^–^23^ and anticipated in theory as triply or doubly hydrogen-bonded dimers,^24^,^25^ phosphate oligomerization is rare in solution. Like charges in anion–anion species display long-range repulsions, not attractions. Perchlorate fulfills this coulombic expectation (Fig. 1a). Nevertheless, such anion–anion oligomers have opened up a new mode of recognition now being specifically investigated with terephthalates,^26^–^30^ bisulfate,^31^,^32^ and most recently with phosphate.^15^,^19^,^20^ These hydroxyanion species are partially stabilized by short-range OH···O hydrogen bonds.^24^,^25^,^33^–^37^ Evidence for them in solution was shown from NMR peak positions (∼13 ppm) of the hydrogen bonded bisulfate dimer [HSO_4_···HSO_4_]^2–^ when further stabilized as complexes^31^ with cyanostar (**CS**) macrocycles (Fig. 1b). While bisulfate dimerizes, the dihydrogen form of phosphate (H_2_PO_4_^–^) bears divergent hydrogen bond donor (–OH···X^–^) and acceptor (–O^–^···HX) sites to enable oligomerization (Fig. 1a). Phosphate oligomerization has been seen in solution^38^,^39^ even without receptors and was previously modelled^14^ in acetonitrile as a simple monomer–dimer equilibrium (*K*_dimer_ = 2400 M^–1^) where 66% of the phosphate is dimerized at 1 mM. Presumably, the propensity of phosphate to oligomerize as well as the characteristics of the receptors will help determine the types of complexes it forms. We test these ideas here using stackable cyanostar macrocycles that can let the phosphates oligomerize into linear assemblies upon complexation.

Complexation of multiple phosphates has only a few examples in solution. Most examples seen in the solid state^6^–^8^,^11^–^23^ fall apart into 1 : 1 complexes^8^,^12^ or were difficult to verify in solution;^6^–^8^,^11^,^15^,^17^–^23^,^40^–^42^ until recently. Sessler used one large, enclosed cavity in a bis-calix[4]pyrrole to bind two dihydrogen phosphates. The crystal structure shows phosphates bridged by one water molecule.^15^ Kubik used two cyclic pseudopeptides^19^ defining a cavity inside which was bound a trimer of phosphates, also bridged by water molecules in the crystal.^19^ The corresponding 1 : 2 and 2 : 3 receptor : anion stoichiometries were retained in solution. Kubik also showed a tetramer of phosphates corralled into a cycle by dint of their stabilization inside a large cyclic cavity.^20^ Previous work in this laboratory used a naphthyl-substituted phosphate to direct formation of 2 : 2 complexes with cyanostar involving a phosphate dimer.^43^ These results suggest that phosphate oligomerizes when it has the steric freedom to do so, and the extent of oligomerization can be circumscribed by the size and shape of the receptor's cavity.

The self-recognition properties of cyanostar macrocycles present a unique environment in which to interrogate the stabilization of hydroxyanion oligomers of phosphate. The anion-binding cavity of cyanostar and its propensity to self-associate by stacking (*K* = 225 M^–1^ in dichloromethane, 600 M^–1^ in acetonitrile)^32^ can create an extended tube-like space to stabilize linear phosphate oligomers. This anticipated oligomerization can be best contrasted to the now well studied^31^,^32^ and simpler bisulfate, which only forms a dimer with cyanostar macrocycles. While co-assembly of multiple phosphates and multiple receptors has been observed, *e.g.*, 4 : 4 stoichiometry,^7^,^11^,^19^,^20^ their mutual interactions have not been studied. Previously we studied these interactions with complexes of the bisulfate dianion dimers and showed stabilization benefits from ion pairing in non-polar solvents (chloroform) to form 2 : 2 : 2 receptor : anion : cation complexes. In more polar solvents (acetonitrile mixtures), which enhance π–π stacking, three stacked cyanostars increased the cavity's electropositive potential to form a 3 : 2 species.^32^ In solvent mixtures with methanol (MeOH) present as a competitive solvent, bisulfate dimers were destabilized leading to the formation of 2 : 1 sandwiches typical of anions like ClO_4_^–^.^44^ While we expect these same driving forces to be in play with phosphate, their impact on the recognition cannot be predicted; different outcomes are actually seen to emerge.

Our findings show that phosphate oligomerization, in contrast to bisulfate dimerization,^31^,^32^ induces formation of higher order anion–anion oligomers with cyanostar stacks. One crystal structure shows a 4 : 3 : 3 ratio between a tetrameric stack of cyanostars, a trimer of phosphate anions, and three tetrabutylammonium (TBA^+^) cations for charge balance. In solution, the 4 : 3 cyanostar–phosphate assembly, as well as 3 : 2, 3 : 3 and 2 : 2 co-assemblies, are identified from equivalence points paired with unique ^1^H NMR signals associated with the different stacking modes, *i.e.*, cyanostar tetramer, trimer, and dimer. We see a distribution of species (low fidelity) in all solvent systems examined. This feature emphasizes phosphate's almost pathological tendency to oligomerize and cyanostar's ability to let it happen. Phosphate's unrestricted co-assembly with cyanostar also marks just one of the many specific differences relative to bisulfate's truncation as anion–anion dimers. Unlike bisulfate, co-assemblies of cyanostar stacks and phosphate oligomers are resistant towards ion pairing in chloroform, resist falling apart upon dilution in dichloromethane–acetonitrile, and resist competitive solvation by addition of methanol to dichloromethane. We also demonstrate co-assembly in the solid state with a 1D chain that is composed of a rare repeat of six phosphates and one phosphoric acid (H_3_PO_4_). Ultimately, it is the phosphate and its propensity to oligomerize that marshals cyanostar stacking to enable the linear organization of the co-assemblies. Nevertheless, the receptor's stabilizing electropositive pockets with their shapes and sizes dictate both the extent and geometry of the phosphate oligomers.

## Results and discussion

### Solid state structures of stacked cyanostars with dihydrogen phosphate trimers

**Fig. 2:**
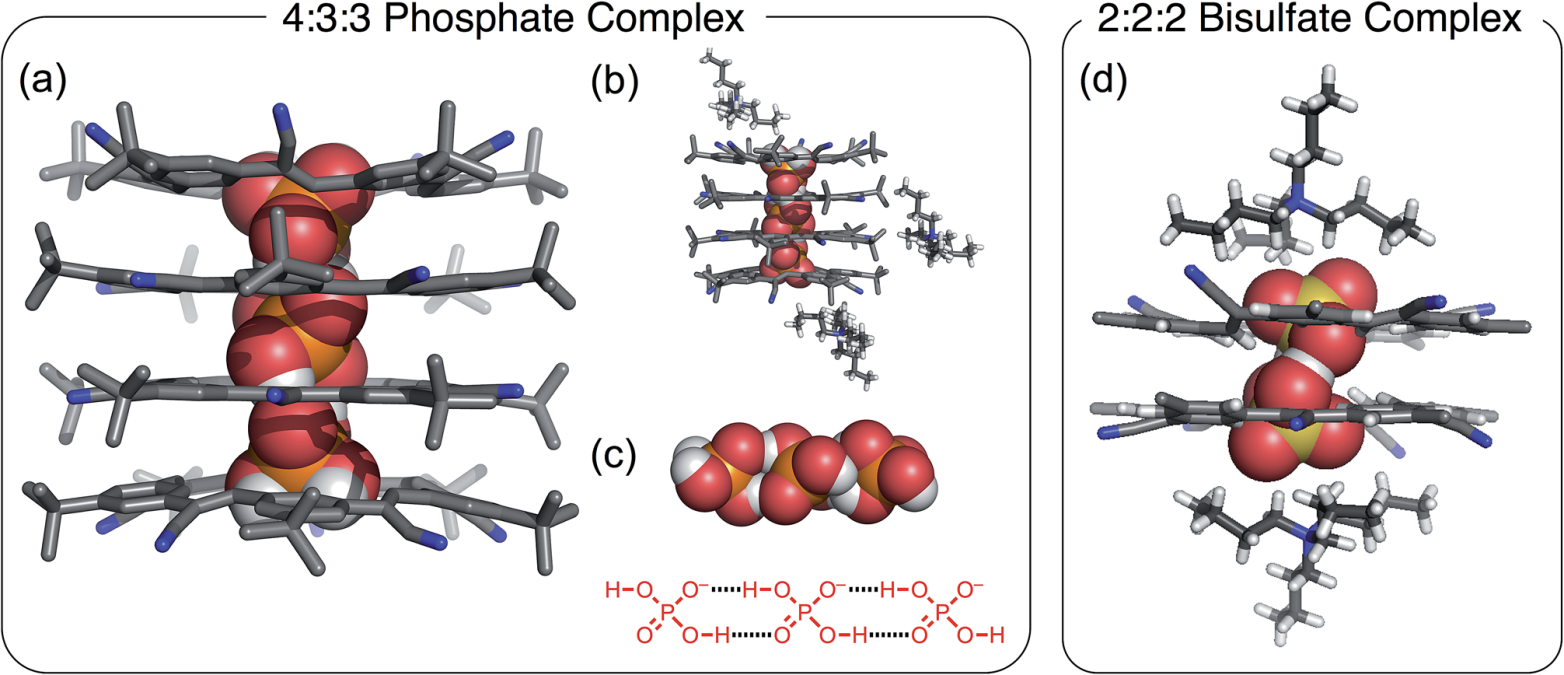
Crystal structure of the 4 : 3 : 3 [**CS**_4_(H_2_PO_4_)_3_(TBA)_3_] showing (a) stack of tetrameric cyanostars with a trianionic tri-phosphate [H_2_PO_4_···H_2_PO_4_···H_2_PO_4_]^3–^ (CCDC ; 1588590); (b) representations highlighting the location of the TBA^+^ cations around the tetrameric stack, and (c) the encapsulated trianionic trimer of phosphates. (d) Crystal structure of the 2 : 2 : 2 bisulfate complex. Crystals were grown by slow diffusion of diethyl ether into saturated chloroform solution of a 1 : 1 : 1 ratio of **CS** : H_2_PO_4_^–^ : TBA^+^.

The crystal structure of cyanostar with tetrabutylammonium (TBA^+^) phosphate (Fig. 2) shows an unprecedented 4 : 3 : 3 stoichiometry for **CS** : H_2_PO_4_^–^ : TBA^+^. This stoichiometry emerges despite growing the crystals from a solution containing an equimolar ratio of host, anion and cation from which a 2 : 2 : 2 assembly was expected based on the bisulfate precedent (Fig. 2b).^31^ This difference in solid-state stoichiometry highlights phosphate's preference for oligomerization over bisulfate's simpler dimerization.

The cyanostar–phosphate complex (Fig. 2) shows a stack of four cyanostars threaded by three dihydrogen phosphate monoanions all hydrogen bonded together as a trianionic trimer [H_2_PO_4_···H_2_PO_4_···H_2_PO_4_]^3–^. The inter-phosphate O···O distance of 2.57 Å (average of all four values) is indicative of borderline strong hydrogen bonds.^45^ These distances are consistent with other oligomeric phosphate species and are similar to the 2.51 Å hydrogen-bond distance seen between bisulfate anions within the cyanostar complex of [HSO_4_···HSO_4_]^2–^.^31^ As with the bisulfate dimer, the anion phosphate trimer is not expected to be stable as a result of coulombic repulsions. To mitigate this long-range repulsion, an alternative sequence of phosphate species was considered in which the central species was a neutral phosphoric acid instead of an anion. However, the 4 : 3 : 3 ratio involving the phosphate trimer was verified from the 4 : 3 intensity ratio seen in the proton peaks in the ^1^H NMR signals of the cyanostar and cation signals acquired from the crystals dissolved in solution (chloroform, Fig. S1†).

Structurally, this crystal structure provides the first evidence that cyanostar macrocycles can be flat. The inner pair of macrocycles within the tetrameric stack are flat. While unusual, these cyanostars reflect the previously characterized conformational energy landscape, which was found to have many shallow minima.^46^ The inter-macrocycle distances between all macrocycles are 3.6 Å (based on centroids defined by the inner phenylene carbon atoms). This distance is similar to those seen in other cyanostar structures,^31^,^43^,^44^,^46^–^49^ which all take the shape of shallow bowls as do the two outermost macrocycles in the tetrameric stack. Each macrocycle displays the whole-molecule disorder seen elsewhere.^31^,^43^,^44^,^46^–^49^ This disorder is associated with the isosteric placement of macrocycles with either *M* or *P* bowl chirality onto identical lattice sites during crystal growth.

The crystal structure shows that the phosphate oligomers do not gain as much stability from the counter cations as was the case with the bisulfate dimer. The crystal structure (Fig. 2b) shows two of the three TBA^+^ cations situated near the top and bottom of the stack, however, they are not in intimate contact with the phosphate trianion. The shortest CH···O contact (4.4 Å) between cation and anion is significantly longer than the sum of van der Waals distances of constituent atoms (H···O) ∼2.7 Å as well as being longer than the corresponding contact in the bisulfate structure (2.4 Å, Fig. 2d). Unlike bisulfate, which truncates as the dimer to engage a stacked cyanostar dimer, the phosphate prefers to oligomerize to a trimer. This trianion thereby recruits two more cyanostars that, as shown previously,^32^ enhances the electropositive character of the cavity. We surmise, therefore, that more stability is gained by combining the phosphate dimer with one extra anion and two more cyanostar macrocycles than the phosphate dianion dimer would gain from tight pairing with two cations in a 2 : 2 : 2 stoichiometry. On the basis of this idea, we investigated different crystal growing conditions to instead try and tip the balance in favor of ion pairing and formation of the 2 : 2 : 2 crystal.

### Increasing ion pairing produces phosphate-based chains in the solid state

Unexpectedly, the addition of extra TBA^+^ cations to the crystal-growing solution leads to the formation of a molecular chain composed of phosphate anions interspersed with an occasional phosphoric acid.^6^–^8^ In this case, additional TBA^+^ cations were added in order to increase the driving force for ion pairing. On account of the fact that cations are always accompanied by anions, we needed to add the TBA^+^ as the salt of a weakly coordinating anion. For this purpose, we selected salicylate^50^ and tris(pentafluorophenyl)borane (BArF) anions because they are too large to complex with cyanostar. These salts were added to the crystal growing solution in order to increase ion pairing. Crystals were grown from a 1 : 1 mixture of cyanostar and the phosphate salt (TBAH_2_PO_4_) with an additional one equivalent of TBA^+^ as the salicylate salt. To validate the generality of the molecular chain in the solid state, crystals were also grown using the equally weak BArF anion; the same crystal structure was obtained. Interestingly, neither the salicylate nor BArF anions are incorporated into the crystal, and thus, we assume that they remain in the mother liquor.

The stoichiometry in the unit cell is consistent with a ratio of four cyanostars, six phosphates, one phosphoric acid, and six charge-balancing TBA^+^ counter cations; 4(**CS**) : 6(H_2_PO_4_^–^) : 1(H_3_PO_4_) : 6(TBA^+^). The acid likely emerges on account of its modest acidity, p*K*_a1_ = 2.1 (water). Consistently, the acid form is not seen with cyanostar complexes of bisulfate, which is attributed to sulfuric acid's strength, p*K*_a1_ = –3 (water).

**Fig. 3:**
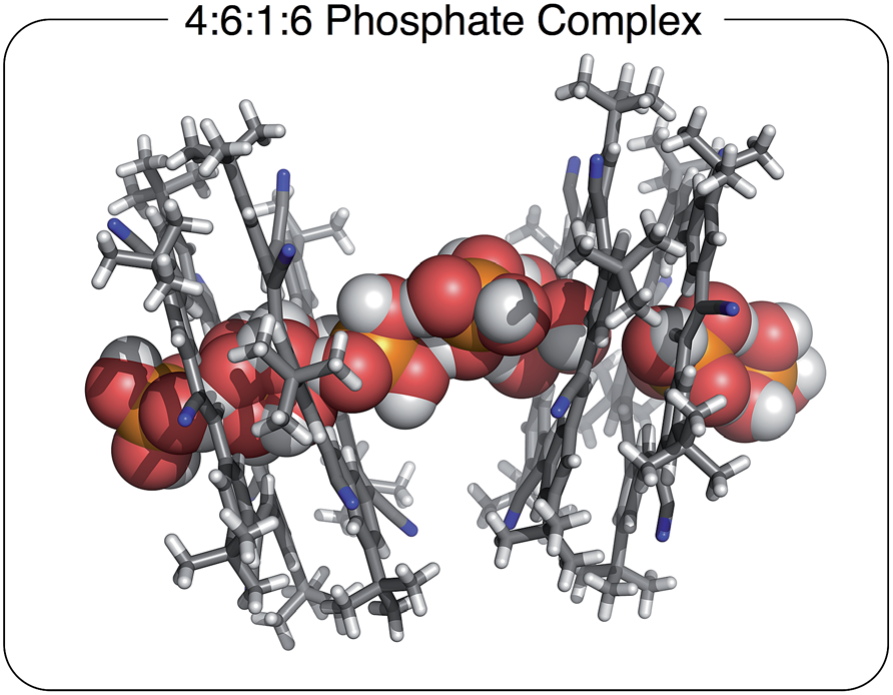
Solid state structure of [**CS**_4_(H_2_PO_4_)_6_(H_3_PO_4_)(TBA)_6_] with a 4 : 6 : 1 : 6 stoichiometry grown in the presence of excess TBA^+^ cation. Chloroform solvent molecules removed for clarity (CCDC ; 1588591).

The 4(**CS**) : 6(H_2_PO_4_^–^) : 1(H_3_PO_4_) : 6(TBA^+^) crystal structure observed with the extra cations (Fig. 3) is significantly different from the 4 : 3 : 3 species (Fig. 2). This structure is characterized by molecular chains dominated by phosphate anions, six for every one phosphoric acid, running continuously through the entire crystal. The chains alternate between being either threaded through and stabilized by cyanostar dimers or paired with TBA^+^ cations in an area shared with solvent molecules. Along the chain, the phosphorous-to-phosphorous distances mostly center at 4.1 Å, similar to the 4.2 Å seen in the 4 : 3 : 3 crystal. There is one phosphorus-to-phosphorus distance of 3.7 Å, which is substantially shorter than the others. Such short distances have been commonly observed in metal-phosphate clusters.^51^,^52^ The phosphate species are interconnected by hydrogen bonding. However, each phosphate species is disordered with all of the refined bond distances averaged and lying between 1.48 and 1.58 Å. Thus, it is difficult to directly distinguish the one phosphoric acid (H_3_PO_4_) from the six phosphates (H_2_PO_4_^–^).

An NMR spectrum (CDCl_3_) of these crystals (Fig. S2 and S3†) shows an aromatic signature that most closely matches a 2 : 2 cyanostar : phosphate species. For comparison, see the NMR titration data conducted in chloroform (*vide infra*, Fig. 5). There is a small resonance at ∼13 ppm seen from solutions of the crystals grown with the salicylate salt (Fig. S3†). This 13 ppm signal is assigned to hydrogen bonded dimers of the phosphate dianionic dimer [H_2_PO_4_···H_2_PO_4_]^2–^. This resonance provides evidence of the self-complementary hydrogen bonding within the solution-phase complexes. In addition to the 2 : 2 complex, we assume that there must be an extra equivalent of TBAH_2_PO_4_ and 0.5 eq. of phosphoric acid present in solution.

### Recognition behavior of cyanostar with phosphate in solution

**Fig. 4:**
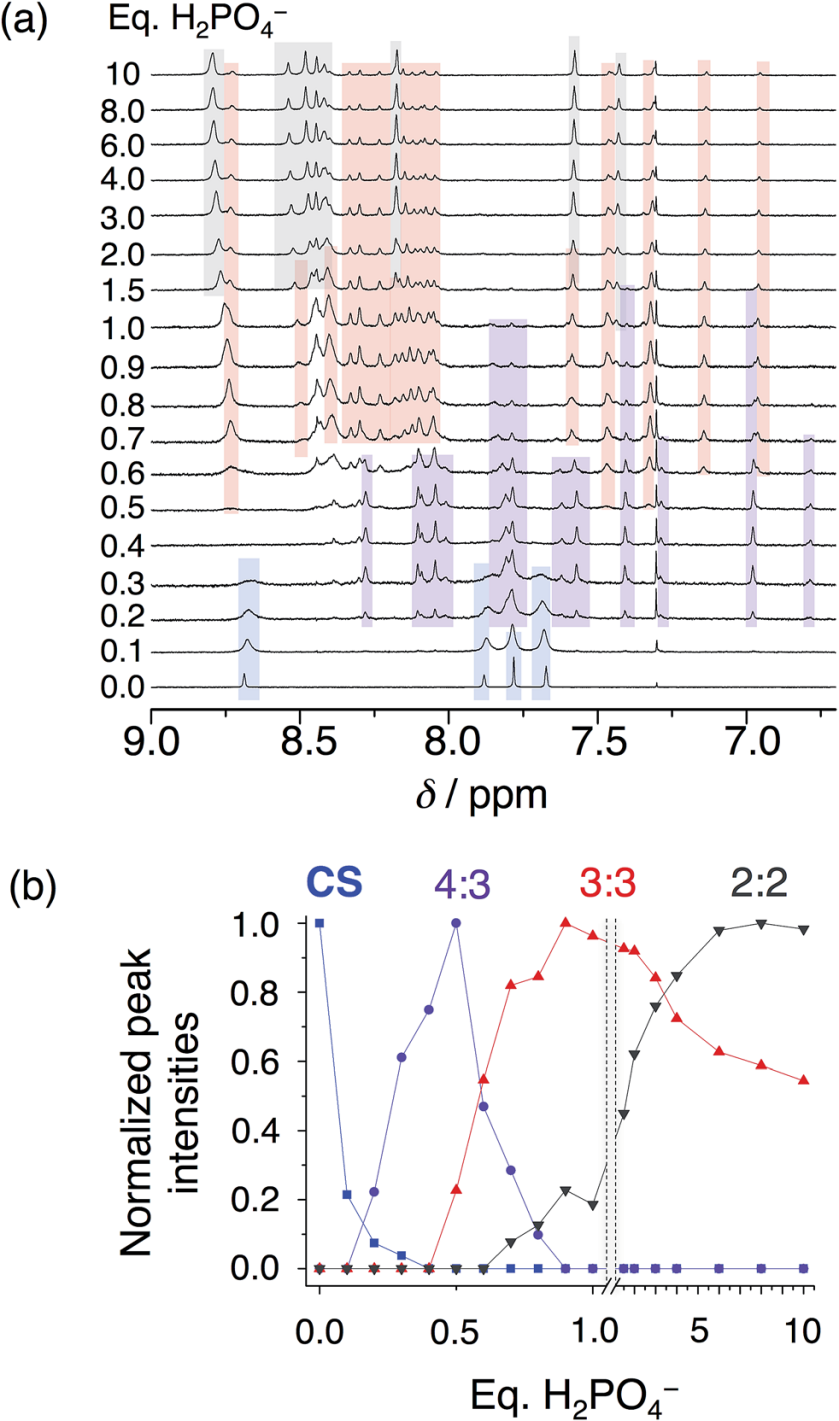
(a) Titration of cyanostar (1 mM) with H_2_PO_4_^–^ in dichloromethane (298 K, 500 MHz). (b) Plot of intensities of peaks belonging to the 4 : 3 (6.78 ppm), 3 : 3 (6.96 ppm), and 2 : 2 species (7.43 ppm), as well as the parent cyanostar (8.69 ppm).

In solution, as with the solid state, phosphate oligomerization generates very different behavior than that observed with bisulfate. We can see 4 : 3, 3 : 2, 3 : 3, and 2 : 2 cyanostar–phosphate co-assemblies depending on solvent. The ^1^H NMR titrations conducted in dichloromethane (Fig. 4) reveal clear aromatic signatures of the stack states. The addition of phosphate to cyanostar shows (Fig. 4a) the species form and disappear broadly along the lines of mass balance (Fig. 4b). We assigned the various peaks to specific species based on the similar signatures seen previously for the triple stack and double stack complexes formed with bisulfate.^31^,^32^ In addition to these, the newest member is assigned to the tetrameric stack with a key marker band at 6.78 ppm. This resonance is more upfield than the 6.96 ppm peak of the triple stacked species. Thus, the 6.78 ppm peak likely arises from a hydrogen on the two inner macrocycles located deeper inside the π-stacked environment of the tetrameric species.

On the basis of our assignments, we see (Fig. 4b) the free cyanostar converts first into the tetrameric stack assigned to a 4 : 3 species with addition of phosphate. We see the 4 : 3 peak intensities maximize at 0.5 eq. and then get consumed by 0.9 eq. At higher concentrations, host–host interactions are enhanced and we see the 4 : 3 species maximize at 0.7–0.8 eq. As the titration proceeds, we see the triple stacked co-assembly with phosphate that we assign to be a 3 : 3 species. The assignment of the triple stack to the 3 : 3 instead of 3 : 2 stoichiometry is based on its place in the sequence of mass balance and its prevalence in the mass spectrometry as a 3 : 3 : 1 and even some 3 : 4 : 2 monovalent species (see Fig. S4†). There is also some 3 : 2 suggesting that it cannot be completely excluded as a possible species. Clearly the ion pairing enhances the tri and tetra phosphate assemblies inside the trimeric stack of cyanostars. Beyond this stage in the titration, the 2 : 2 co-assembly is seen to be formed and to keep climbing in intensity. Low fidelity speciation between each of the co-assemblies is seen across the entire titration.

**Fig. 5:**
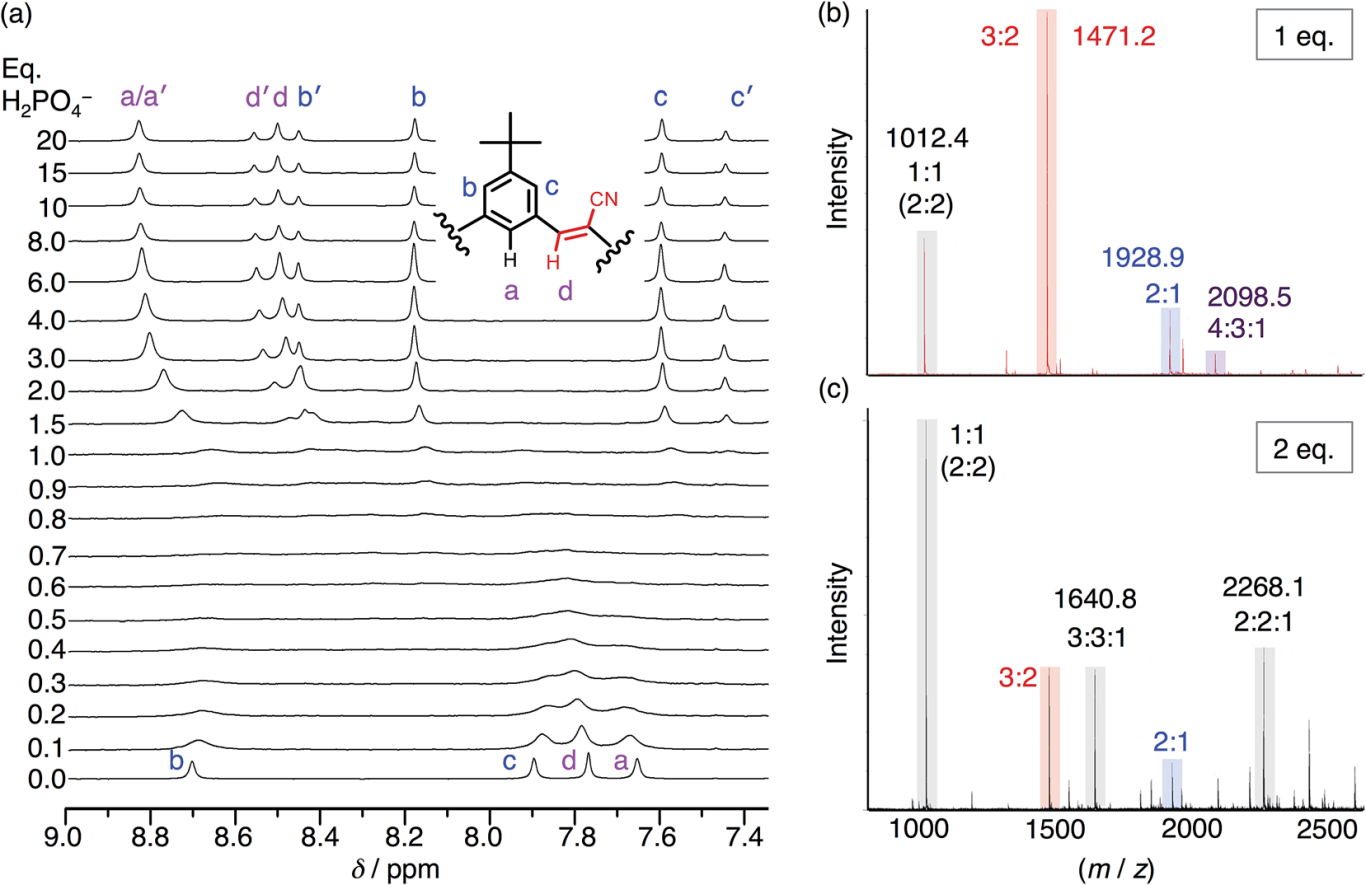
(a) ^1^H NMR titration spectra of **CS** (1 mM) with the addition of H_2_PO_4_^–^ in CDCl_3_. (b) ESI-MS of **CS** (1 mM) with 1 eq. TBAH_2_PO_4_ and (c) 2 eq. TBAH_2_PO_4_ (20 V, 50 °C source).

To help overcome the low fidelity, prior studies with bisulfate^14^ suggested that the low polarity of chloroform (*ε* = 4.5) might drive formation of a 2 : 2 : 2 species as a consequence of enhanced ion pairing. While the NMR titration with phosphate in chloroform (Fig. 5a) ultimately shows formation of the expected 2 : 2 signature, it only emerges later in the titration. This behavior again differs from bisulfate's high-fidelity formation of the 2 : 2 : 2 in chloroform directly at 1 eq. We assign the signature seen with phosphate to a 2 : 2 : *x* species where *x* is an undefined number of counter cations (*x* = 0, 1, 2). Examination of the cation's NMR signals during the titration as well as from a measurement of its diffusion coefficient at 1 eq. (Fig. S5†) shows that the cation has moderate-to-high participation with the cyanostar–phosphate species. We see the 2 : 2 : *x* signature rise from the exchange-broadened baseline around 1.0 eq., and by 2 eq. the signature can be seen most clearly. The inner cyanostar protons of the 2 : 2 : *x* complex are more downfield shifted compared to the corresponding bisulfate complexes. This last observation is consistent with the higher electrostatic potential of the phosphate anion; the electronegativity difference between phosphorus and oxygen is greater than sulfur and oxygen. The correlation between charge density and ^1^H NMR peak position is not perfect,^53^ however, it may be reasonable for these two similarly sized anions.

Even though chloroform was selected to enhance ion pairing, phosphate instead continues to express its propensity to oligomerize. In further support of that idea, the NMR spectrum (Fig. 5a) recorded from 0.3–1.0 eq. shows broadened signals attributed to exchange between multiple higher-order species. One of the possible species can be identified in the electrospray-ionization mass spectrometry (ESI-MS). With 1 eq. of phosphate (Fig. 5b), the spectrum is dominated by the dianionic 3 : 2(2–) species (*m*/*z* 1471.2 Da) with a few other minor peaks. It is only with the addition of 2 eq. (Fig. 5c) that the 3 : 2 species gets outcompeted in favor of various species that each share an equimolar ratio between cyanostar and phosphate plus the occasional counter cation, *i.e.*, 1 : 1(1–), 2 : 2(2–), 3 : 3 : 1(2–) and 2 : 2 : 1(1–). Furthermore, the signature for the tetrameric and trimeric stacks are seen in ^1^H NMR titrations conducted at 10 mM (Fig. S5†).

### Effects of phosphate solvation on the co-assemblies with cyanostar

**Fig. 6:**
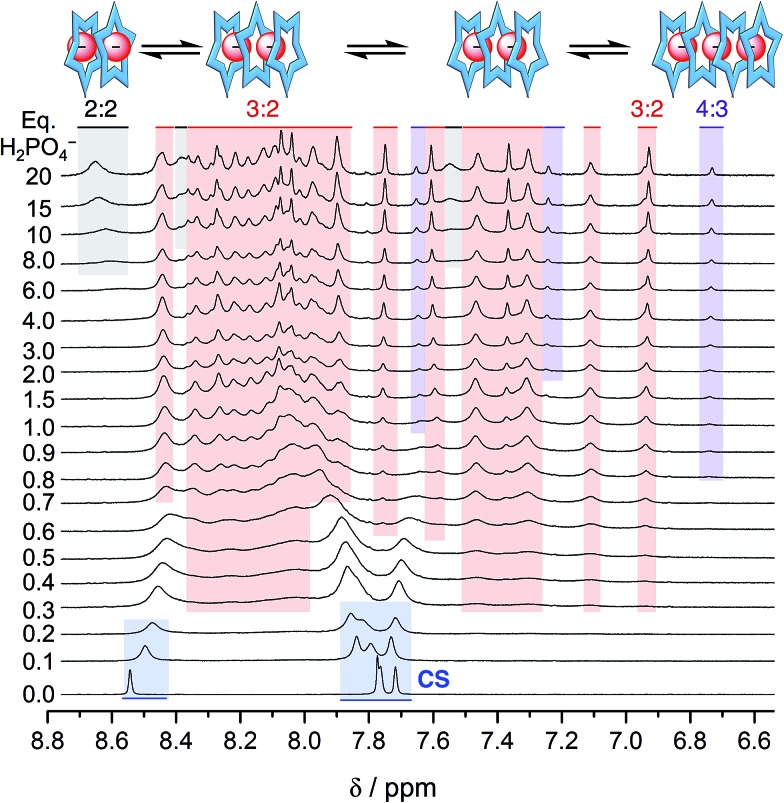
^1^H NMR spectra of the titration of cyanostar (10 mM) with H_2_PO_4_^–^ in 60/40 v/v% CD_2_Cl_2_/CD_3_OD.

Surprisingly, the phosphate's oligomerization can even outcompete the strong solvation of hydroxyanions that normally accompanies use of protic hydroxy solvents, such as methanol. Competitive solvation of the hydroxyanion was particularly striking with bisulfate,^14^ where we observed direct and exclusive formation of the 2 : 1 species in a methanol–CH_2_Cl_2_ mixture (40/60 v/v%). We believe that molecules of methanol partially solvate the bisulfate anion inside the cyanostar complex. With phosphate, however, use of methanol as a co-solvent does not truncate the anion's oligomerization. Rather, the titration data (Fig. 6) indicates the 4 : 3 and 3 : 2 species are produced without loss of anion–anion self-assembly. Specifically, early on we see migrations indicative of formation of a small amount of the 2 : 1 complex. Then from 0.3 eq. onwards the ∼24-peak signature of the 3 : 2 complex emerges with its characteristic peak at 6.9 ppm. A little later in the titration (>0.6 eq.), we see the marker band for the 4 : 3 species at 6.7 ppm.

The sequence of species follows the expectations of mass balance but it differs from pure dichloromethane in an interesting way. In dichloromethane, the triple stack can support a trimer of phosphates when ion paired with TBA^+^ cations as the 3 : 3 : 1 and possibly 3 : 4 : 2 species. Thus, the triple stacked signature emerges after that of the 4 : 3 species. In the methanol mixture, however, the triple stack can only support a dimer and its signature emerges prior to that of the 4 : 3. This is a case where the polar methanol easily screens a traditional electrostatic stabilization (anion–cation) but the non-traditional electrostatic interactions (anion–anion) does not; the microenvironment of the stacked cyanostars presumably playing a critical role.

**Fig. 7:**
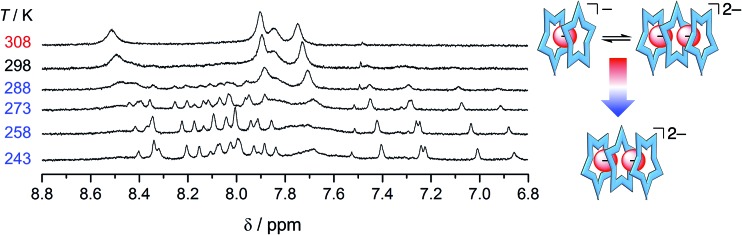
Variable temperature ^1^H NMR spectra of cyanostar (1 mM) with 0.5 eq. H_2_PO_4_^–^ in 60/40 v/v% CD_2_Cl_2_/CD_3_OD.

The resistance of phosphate's oligomerization to competitive solvation is another clear departure from bisulfate. A comparison of the ESI-MS is consistent with the differences between the two anions (see Fig. S8†): bisulfate prefers the 2 : 1 in the methanol solution mixture,^32^ while phosphate retains multiple higher order species. As further corroboration of this idea, we characterized a solution bearing 0.5 eq. of phosphate as a function of temperature (Fig. 7). Upon cooling, we see the signature for the 3 : 2 species clearly emerge from the exchange-averaged spectrum seen at room temperature. Higher order species are expected to be favored at lower temperatures. For comparison, when the same experiment was conducted with 0.5 eq. bisulfate only the 2 : 1 sandwich was observed upon cooling.

### Complexes of phosphate monomers are unfavored

Another unexpected feature emerges in the recognition of phosphate, specifically, it appears there is poor affinity between cyanostars and just one phosphate, *i.e.*, low stability of the 2 : 1 complex. In the methanol mixture, the broadened NMR signature seen below 0.5 eq. is attributed to exchange between free cyanostar and its 2 : 1 complex but the peaks only migrate a little, which is indicative of poor complexation. For example, with 0.5 eq. of phosphate (1 mM, Fig. 7, 298 K) the proton that shifts the most (H_b_) only moves 0.11 ppm, while with perchlorate in the tight 2 : 1 complex it was 0.45 ppm.^44^ Thus, the NMR spectrum shows peak positions that more closely resemble the free cyanostar than the 2 : 1 complex. Consistent with less of the 2 : 1 species in solution, the diffusion NMR recorded in the presence of 0.5 eq. of anion shows a smaller change in the diffusion coefficient of cyanostar with the addition of phosphate than compared to additions of either bisulfate or perchlorate (see ESI, Fig. S9†). We make very similar observations in other solvents suggesting this is a general phenomenon involving phosphate. In dichloromethane (Fig. 4), early peak shifts are negligible and suggest that very little of the 2 : 1 is formed in this solvent. The same is true for 40/60 v/v% acetonitrile/dichloromethane mixture (ESI†).

To further investigate the weak stability of the 2 : 1 phosphate complex, we conducted variable concentration studies. We selected 1 eq. of anion and a 40/60 v/v% methanol : dichloromethane solvent mixture in order to compare our findings to the variable concentration data recorded with bisulfate.^32^ Upon dilution from 10 mM, we see a loss in the peaks at around 6.9 and 6.7 ppm (Fig. S10†), indicating that we are diluting out the higher order 3 : 2 and 4 : 3 species. The resulting aromatic NMR signature from 0.1 mM onwards shows just the free cyanostar instead of the 2 : 1 complex. By comparison the variable concentration study with bisulfate is significantly different. Therein, the 2 : 1 complex seen at mM concentrations is retained even at low concentration (<10 μM).^31^

Taken together, these observations suggest that complexation of phosphate by cyanostar is much greater when the more highly charged dimer dianion and trimer trianion can be formed during the addition of more equivalents of the anion. The behavior observed with phosphate indicates an all or nothing binding: that is, we believe the complexes of the dimers and trimers of phosphate have populations (and stabilities) significantly greater than a single phosphate. Thus, phosphate appears dormant as a guest until it can start to oligomerize. This type of behaviour has been reported elsewhere.^17^

### Effect of solvophobic stacking of assemblies of cyanostar with dihydrogen phosphate

Based on the study with bisulfate,^32^ we expected solvents like acetonitrile to enhance oligomerization even further by driving macrocycle stacking. This situation would be expected to stabilize higher order species. In 40/60 v/v% acetonitrile/dichloromethane, the NMR titration (Fig. S11†) shows little peak movement from 0–0.5 eq. consistent again with the modest stability of the 2 : 1 sandwich complex. With extra phosphate (>0.5 eq.), a complex pattern is produced indicating formation of trimeric 3 : 2 (6.9 ppm) and tetrameric 4 : 3 species (6.7 ppm). Consistent again with greater stabilization of the π-stacked species, the 6.7 ppm peak of the 4 : 3 co-assembly remains even with a massive excess of phosphate (20 eq.), whereas in pure dichloromethane the 4 : 3 species was completely disappeared at 0.9 eq.

**Fig. 8:**
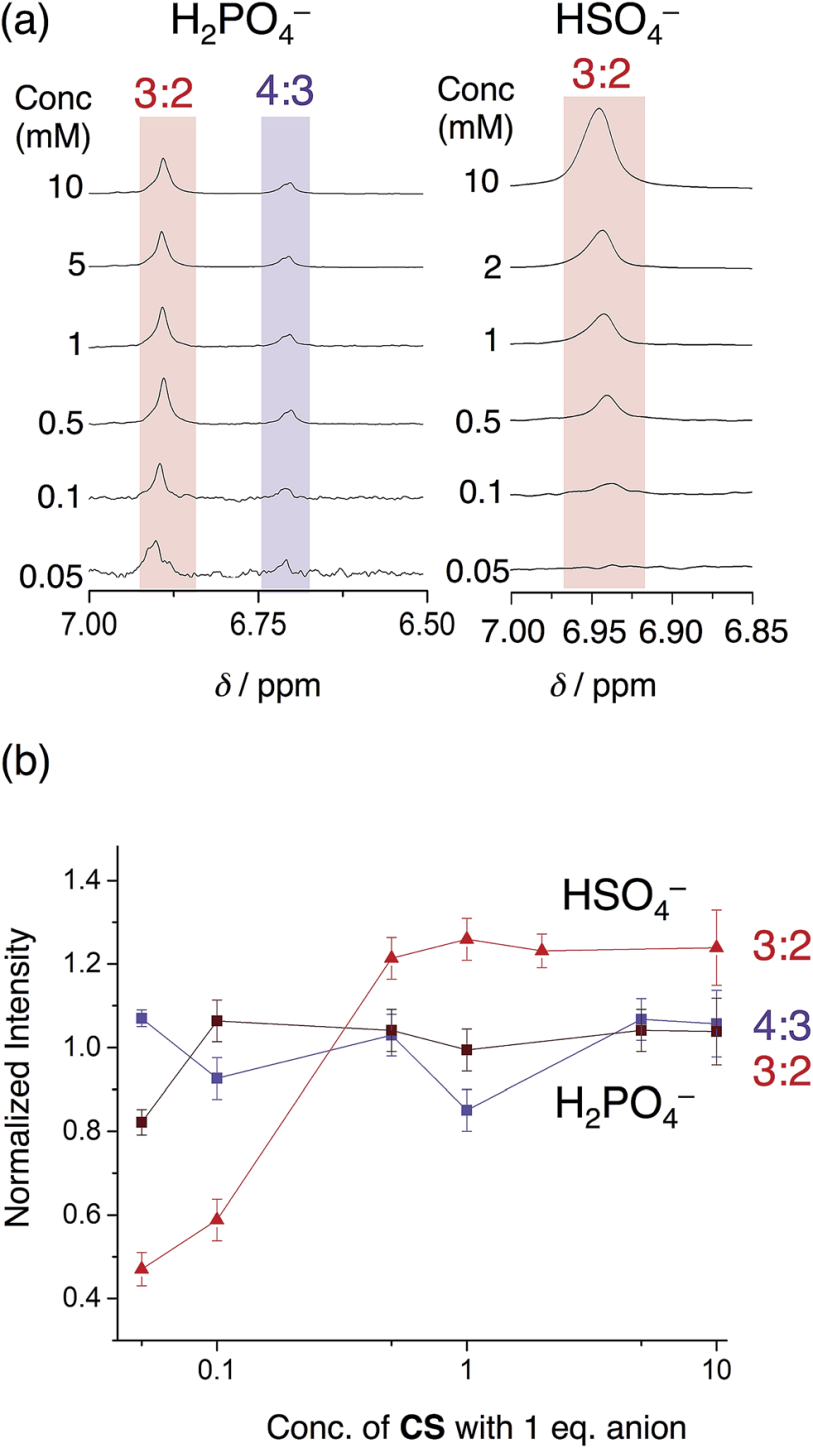
(a) Variable concentration study of cyanostar with 1 eq. H_2_PO_4_^–^ compared to 1 eq. HSO_4_^–^ in 40/60 v/v% CD_3_CN/CD_2_Cl_2_ (298 K, 600 MHz), and (b) the normalized intensities of the proton resonances for the co-assemblies as a function of concentration. The intensities of each peak were normalized to the intensity of the alpha proton of the TBA^+^ countercation at *ca.* 3.0 ppm. The 3 : 2 species with bisulfate (6.93 ppm), the 3 : 2 phosphate species (6.96 ppm) and the 4 : 3 phosphate species (6.73 ppm) were obtained from the ^1^H NMR experiments.

The phosphate-based species are also more robust upon dilution when compared to bisulfate complexes in an acetonitrile–dichloromethane mixture. With 1 eq. of bisulfate, the 3 : 2 complex gave way to the 2 : 1 sandwich complex at relatively high concentrations (500 μM). When using phosphate, however, we see the 3 : 2 and 4 : 3 species are retained down to 50 μM (Fig. 8). This finding shows how the enhanced self-association of the cyanostar in the more polar solvent mixture cooperates with the oligomerization of phosphate to enhance the stability of higher-order species.

### Stoichiometries of phosphate–receptor complexes

**Fig. 9:**
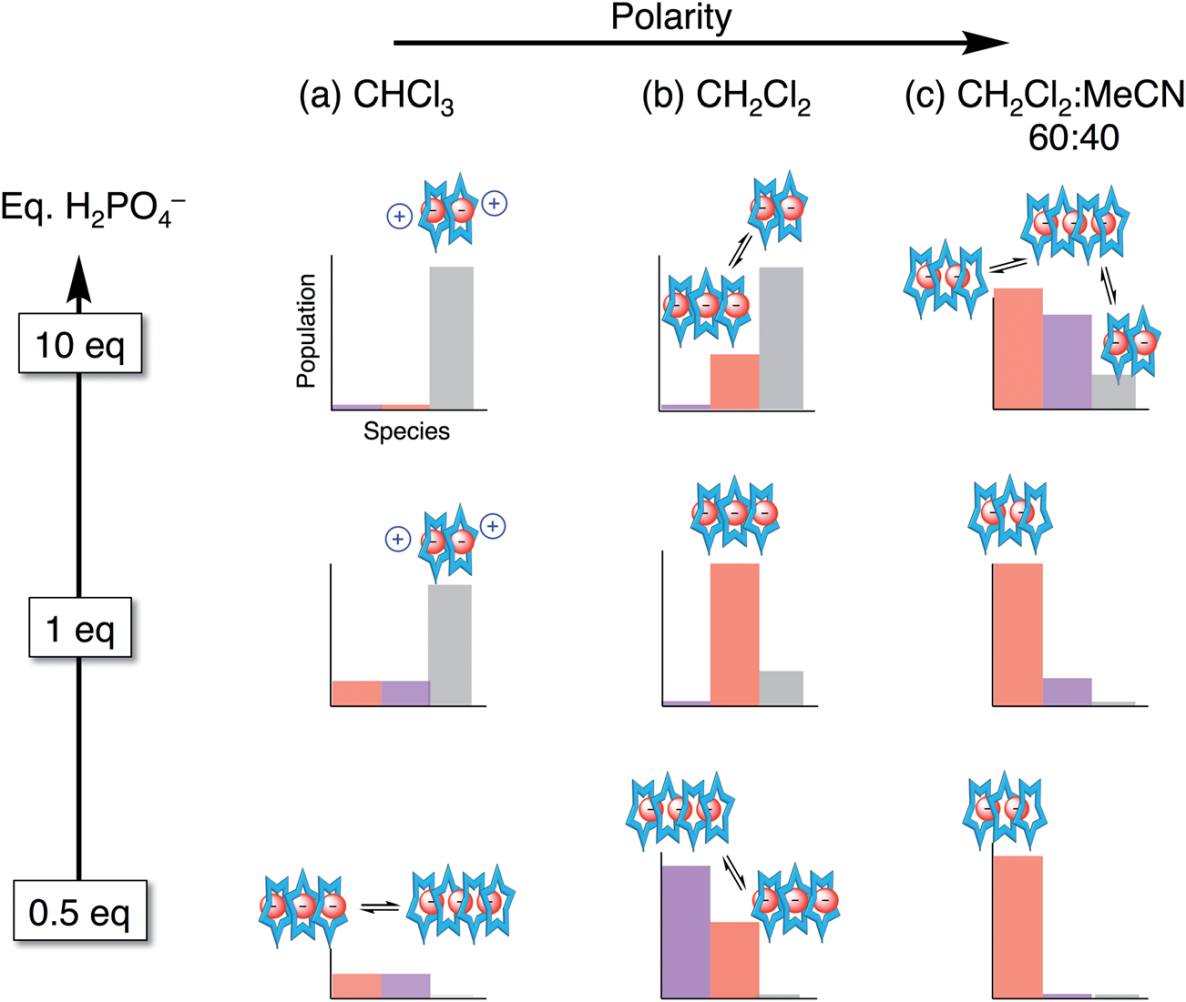
Approximate distribution of species in solvent systems of increasing solution polarity at different stages in the titration.

Overall, we see phosphate oligomerization gets expressed across all solution conditions. In chloroform (*ε*_eff_ = 4.8, Fig. 9a), oligomerization remains despite larger driving forces for ion pairing. In dichloromethane (Fig. 9b), the species observed are believed to be a result of driving forces that favor a mixture of phosphate oligomerization (anion–anion), the cyanostar stacking, and ion pairing (anion–cation). Thus, we observe populations largely maximizing at the mole ratios favouring 4 : 3, 3 : 3 : *y*, and 2 : 2 : *x*. In the methanol and acetonitrile mixtures (Fig. 9c), however, the balance is tipped away from ion pairing and towards π-stacking with higher order species forming and remaining in solution irrespective of the quantity of phosphate added. The extensive oligomerization is seen when cooperating with cyanostar stacking. Its stacking creates a lumen with no end caps (Fig. 1c). This feature was clear in the phosphate–phosphoric acid crystal structure (Fig. 3). Even the counter cations do not serve as effective caps in the solid-state structure. This receptor-templated geometry of the oligomer as a linear chain also matches the behaviors seen with receptors that have convergent shapes: Sessler's bis-calixpyrrole^15^ inhibits anything larger than a dimer and Kubik's receptor^20^ creates a corral that directs the oligomer into a cyclic tetramer.

## Conclusion

Phosphate oligomerization drives the formation of higher order co-assemblies in cooperation with cyanostar. Phosphate trimers [H_2_PO_4_···H_2_PO_4_···H_2_PO_4_]^3–^ inside cyanostar tetramers and trimers as well as phosphate dimers [H_2_PO_4_···H_2_PO_4_]^2–^ inside cyanostar trimers or dimers are observed. Low-fidelity mixtures are seen across various solutions on account of the dominance of oligomerization. The co-assemblies are extremely robust and display behaviors differing significantly from bisulfate in the solid state and in solution. Oligomerization of phosphate is seen to decrease the importance of ion pairing interactions in offsetting the long-range coulombic repulsions emerging when multiple anions are hydrogen bonded to one another. The oligomerization even impacts affinity in a positive manner with a single phosphate appearing to sit dormant until it can oligomerize. We also see that the recognition of anion–anion species is more complex with phosphate than bisulfate on account of its facile protonation and access to phosphoric acid. Ultimately, phosphate's oligomerization is key to understanding its recognition by larger receptors where the shape and size of the receptor appears to control the extent and geometry of the oligomers formed.

## Conflicts of interest

There are no conflicts of interest to declare.

## Supplementary Material

Supplementary information

Crystal structure data
