# Supplementary material for: Phosphate–phosphate oligomerization drives higher order co-assemblies with stacks of cyanostar macrocycles
Source: Chem Sci. 2018 Feb 20;9(11):2863–72. doi: 10.1039/c7sc05290a (PMC5941797; doi:10.1039/c7sc05290a)
Supplement: Supplementary file 1 [file SC-009-C7SC05290A-s001.pdf]

Electronic Supplementary Material (ESI) for Chemical Science.
This journal is © The Royal Society of Chemistry 2018

Electronic Supplementary Information

Chemical Science

Phosphate-phosphate oligomerization drives higher order co-assemblies with stacks of cyanostar macrocycles

Elisabeth M. Fatila, Maren Pink, Eric B. Twum, Jonathan A. Karty, Amar H. Flood

Indiana University, Bloomington, IN, 47405

S1. General methods

Cyanostar was prepared following published procedures.[1] The tetrabutylammonium salts $NBu_4H_2PO_4$ (Sigma Aldrich and Alfa Aesar), and NBu_4HSO_4 were stored in an argon environment prior to use, and NBu_4ClO_4 (TCI America) was used as received. Spectroscopic grade solvents (CH_2Cl_2, CH_3CN, CH_3OH and $CHCl_3$ (Omnisolv)), and deuterated solvents (CD_2Cl_2, $CDCl_3$, CD_3CN and CD_3OD (Cambridge Isotope Laboratories and Sigma Aldrich)) were used as received.

Titrations were monitored by ^1H NMR. All titrations were dilution corrected. ^1H 1D NMR, ^{31}P NMR and ^1H diffusion NMR spectra were recorded on Varian Inova (400, 500 and 600 MHz) spectrometers. Chemical shift positions were determined using solvent residue peaks. In a typical ^1H NMR titration, 500 µL of a **CS** (1 mM) solution was prepared in a screw-cap NMR tube equipped with PTFE/silicone septa. The salt solutions (~50 eq./1 mL) were prepared immediately before use and stored in screw-cap vials equipped with PTFE/silicone septa. Salt solutions were added using Hamilton gas-tight syringes (10, 50 500 µL). High-resolution ESI analyses were performed using a Waters Synapt HDMS time-of-flight mass spectrometer that was calibrated externally with sodium trifluoroacetate clusters over a wide mass range: *m*/*z* 800-3800. The sample was directly infused and data acquired in the negative ion W-mode (resolving power: 16000). The cone voltage was kept constant (20 V) and the source temperature held at 50 °C.

S2. ¹H and ³¹P NMR of Crystals

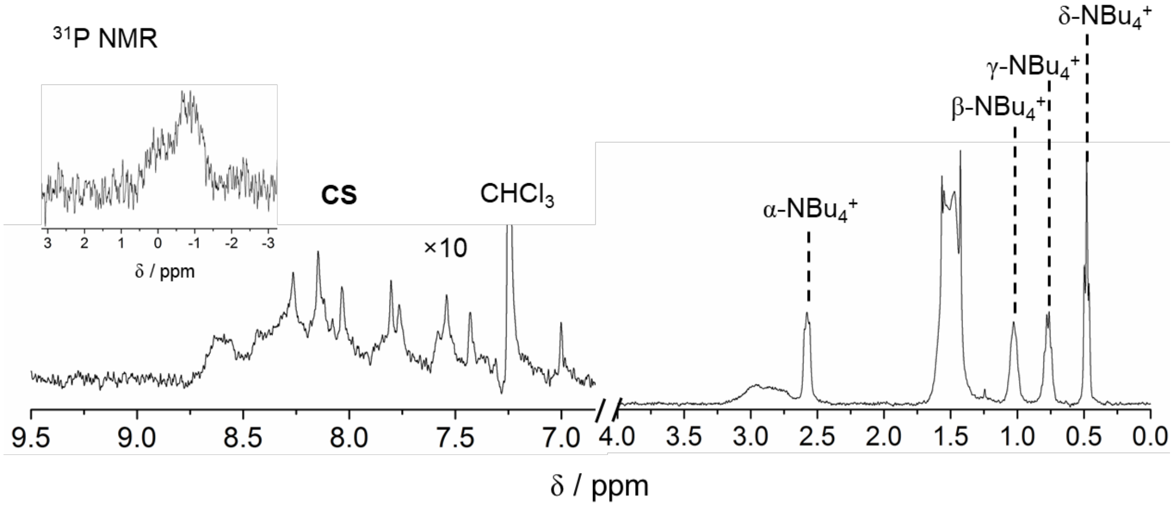

Figure S1. ^{1}H NMR and ^{31}P NMR (inset) of crystals of [**CS**$_4$(H_2PO_4)$_3$(TBA)$_3$] in $CDCl_3$ (298 K, 600 MHz).

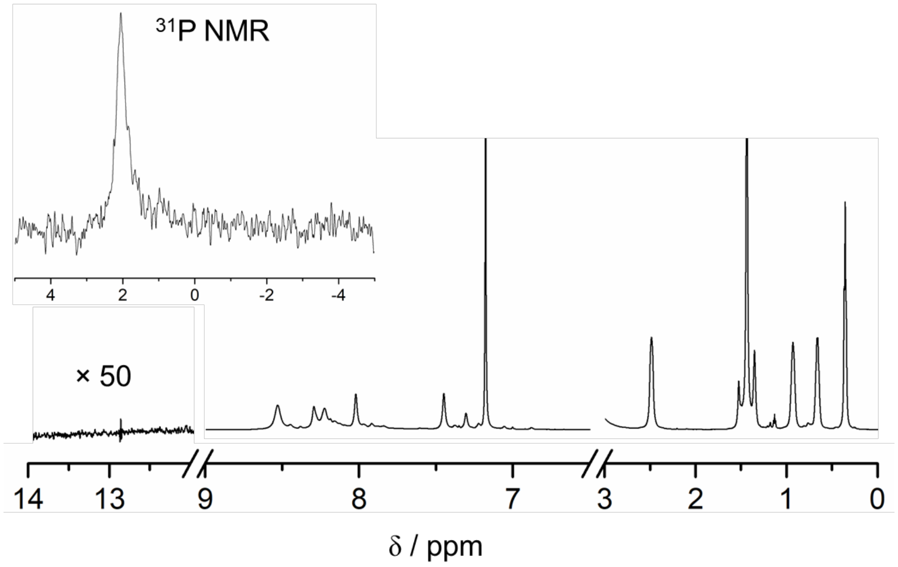

Figure S2. ^{1}H NMR and ^{31}P NMR (inset) of the crystals of [**CS**$_4$(H_2PO_4)$_6$(H_3PO_4)$_1$(TBA)$_6$] grown from TBA(BArF) in $CDCl_3$ (298 K, 600 MHz).

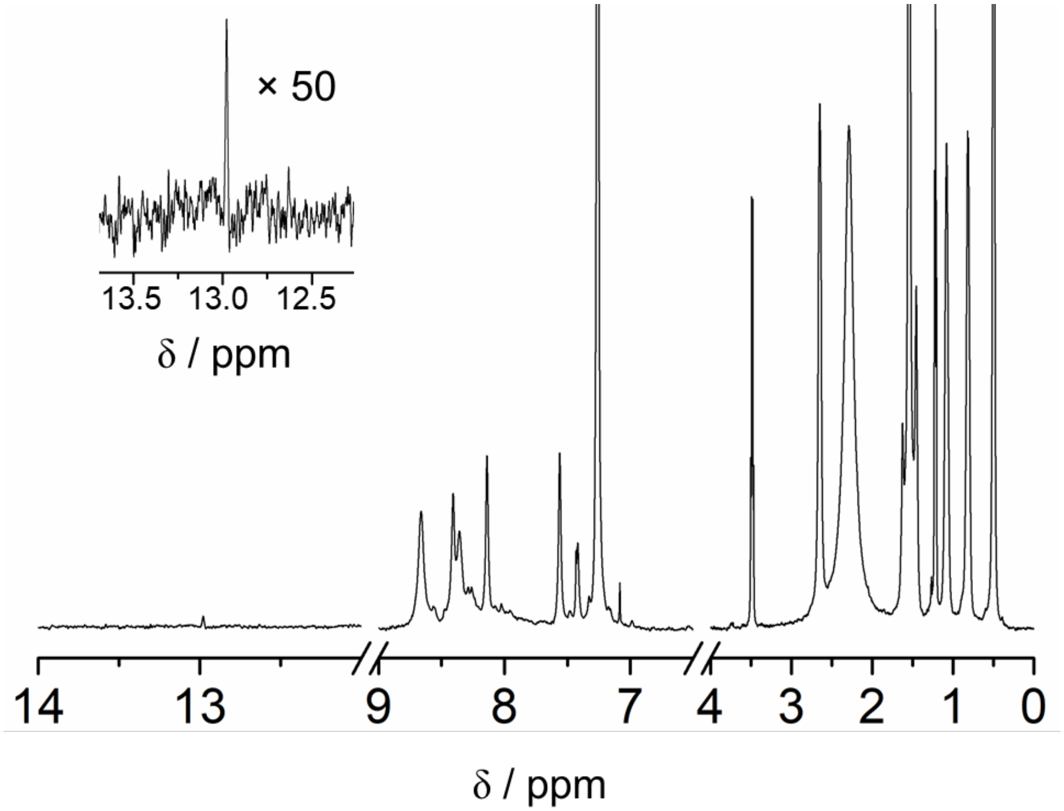

Figure S3. ^{1}H NMR of crystals of [$\mathbf{CS}_4(H_2PO_4)_6(H_3PO_4)_1(TBA)_6$] grown from TBA(salicylate) in $CDCl_3$ (298 K, 600 MHz).

S3. ESI-MS of Cyanostar with TBA(H_2PO_4) in Dichloromethane

The finding that phosphate's oligomerization allows multiple species to emerge in solution is supported by ESI-MS data. Quite independent of the solvent conditions, a rich array of species is observed. Furthermore, and as noted with bisulfate,[2, 3] the electrospray conditions extend the numbers of species beyond what is seen in solution though it is far more extensive with phosphate. By way of example, consider the spectrum obtained with addition of 2.5 eq. of $TBAH_2PO_4$ in CH_2Cl_2 at 2 mM (Figure S4). We see the following species with these **CS**:anion:cation ratios and charge state: 2:2:0 (2–), 4:3:0 (3–), 3:2:0 (2–), 3:3:1 (2–), 3:4:2 (2–), and 2:2:1 (1–) with other peaks also present.

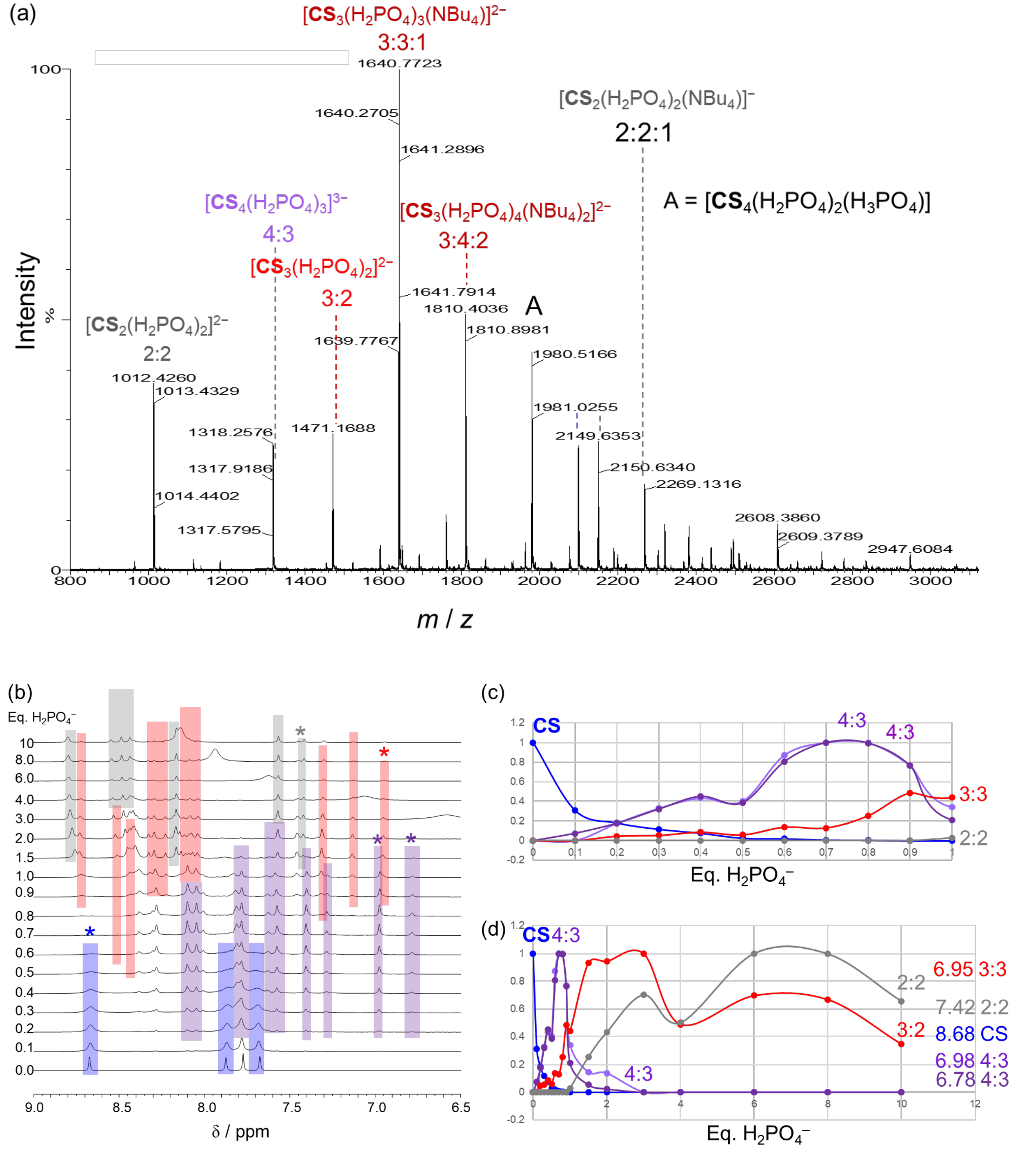

Figure S4. (a) ESI-MS of **CS** (2 mM) with 2.5 eq. $TBAH_2PO_4$ in CH_2Cl_2 (50 °C, 20 V, negative mode). (b) ^{1}H NMR titration of **CS** (5 mM) with $TBAH_2PO_4$ in CH_2Cl_2 (color codes match main text). (c and d) Titration plots (5 mM) using normalized NMR band intensities.

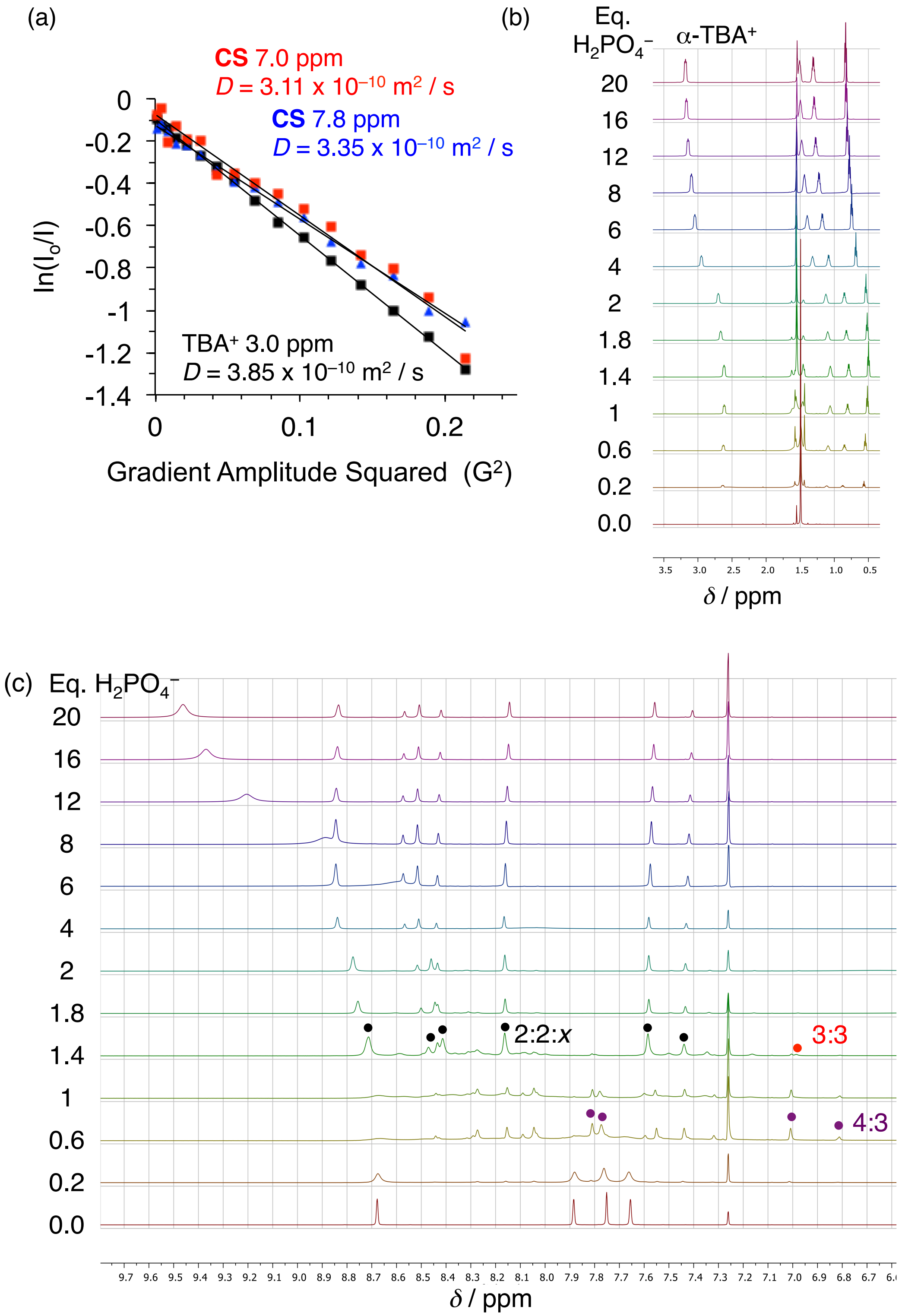

Figure S5. (a) Diffusion gradients data and fitting for **CS** (10 mM), $CHCl_3$, and TBA^+ protons in $CDCl_3$ with 1 eq. $TBAH_2PO_4$ added (600 MHz, 298 K). (b and c) Associated NMR titration (10 mM); with the signature peaks for the 4:3, 3:3 and 2:2:*x* marked. Intensities adjusted to enable visualization.

S4. Ion pairing and self-association of tetrabutylammonium salts

To examine the propensity for self-association without the cyanostar, we conducted a variable concentration study of the tetrabutylammonium salts. From 0–333 mM we see (Figure S5) a similar movement of the H_2O protons as that seen for $TBAHSO_4$. The TBA^+ protons for the $H_2PO_4^-$ salt shift over a wider range compared to the HSO_4^- salt. We also compared the diffusion coefficients (Figure S6) across anions using perchlorate as a non-oligomeric control. First, diffusion values are smaller along the series ClO_4^- > HSO_4^- > $H_2PO_4^-$ consistent with greater extent of oligomerization of phosphate. We see ClO_4^- shows the same diffusion change with concentration as the $TBAH_2PO_4$ and $TBAHSO_4$ salt suggesting changes in viscosity at higher concentrations (>10 mM) control diffusion rather than extensive oligomerization.

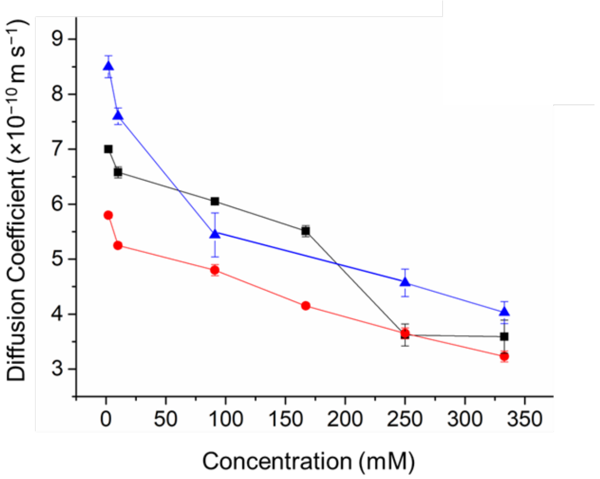

Figure S6. Diffusion coefficient of NMR of $TBAClO_4$ (blue triangles), $TBAH_2PO_4$ (red circles), and $TBAHSO_4$ (black squares) in $CDCl_3$ (298 K, 600 MHz).

S5. ^1H NMR data of CS with $TBAH_2PO_4$ in 60/40 v/v % CD_2Cl_2/CD_3OD

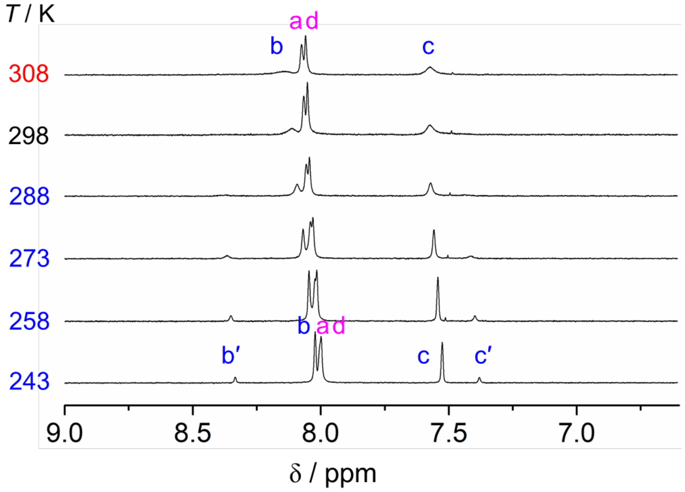

Figure S7. Variable temperature of **CS** (1 mM) with 0.5 eq. $TBAHSO_4$ in 60/40 *v*/*v* % CD_2Cl_2/CD_3OD (500 MHz).

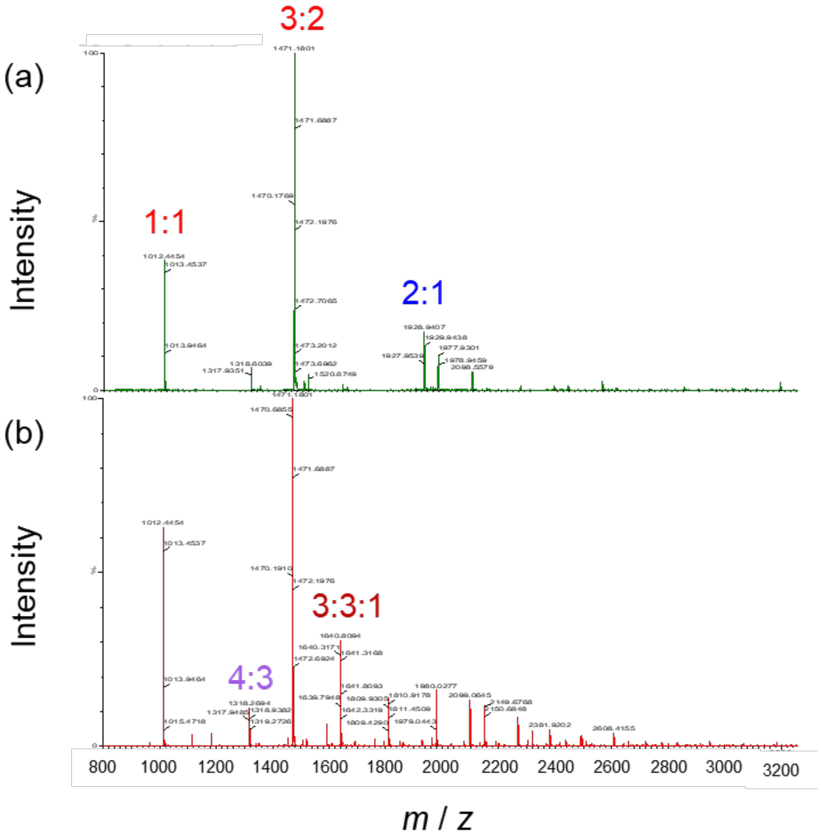

Figure S8. ESI-MS of **CS** with (a) 0.5 eq. $TBAH_2PO_4$ and (b) 1 eq. $TBAH_2PO_4$ in 60/40 *v*/*v* % CD_2Cl_2/CD_3OD (50 °C, 20 V, negative mode).

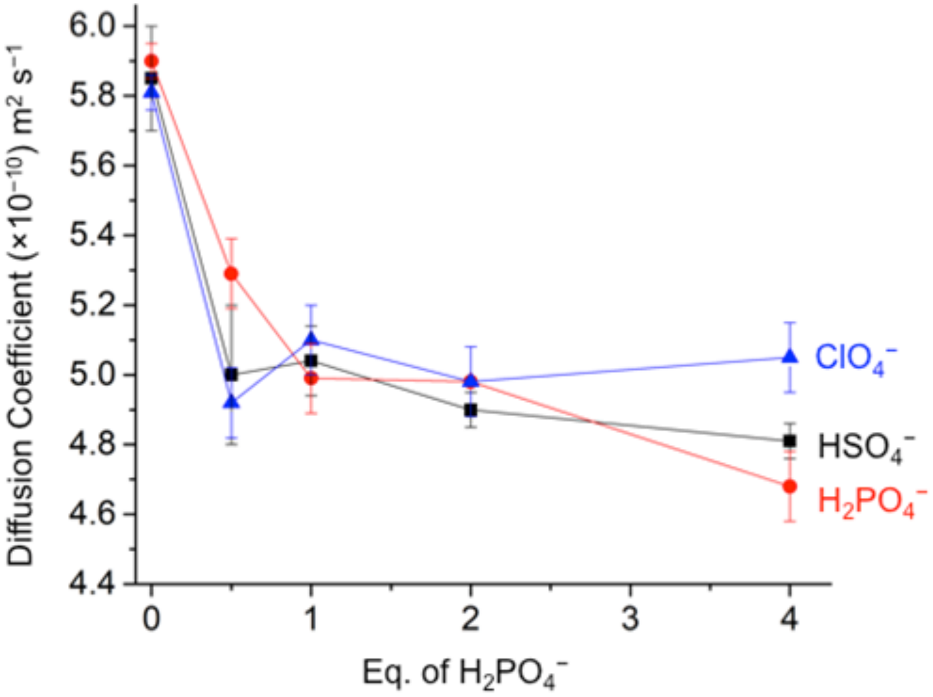

Figure S9. ^{1}H Diffusion NMR of **CS** (1 mM) with various anions as their TBA^+ salts in 60/40 *v*/*v* % DCM/MeOH (298 K, 600 MHz). The aromatic protons and *t*-butyl protons of **CS** were averaged.

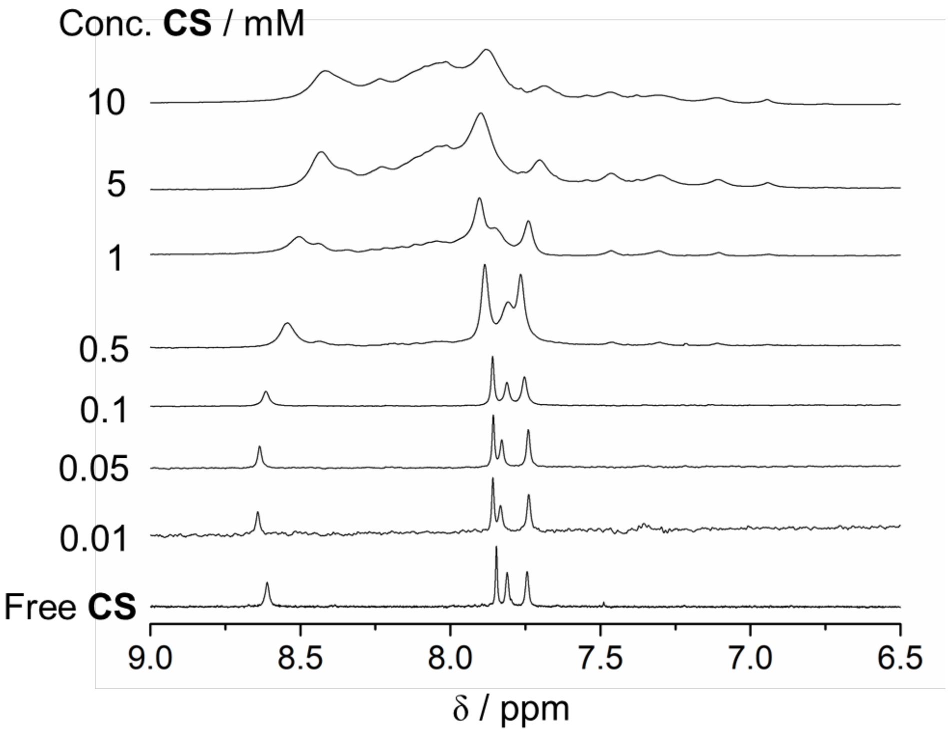

Figure S10. Variable concentration of **CS** with 1 eq. $TBAH_2PO_4$ in 60/40 *v*/*v* % CD_2Cl_2/CD_3OD (600 MHz, 298 K).

S6. Cyanostar with $NBu_4H_2PO_4$ in 60/40 v/v % CD_2Cl_2/CD_3CN

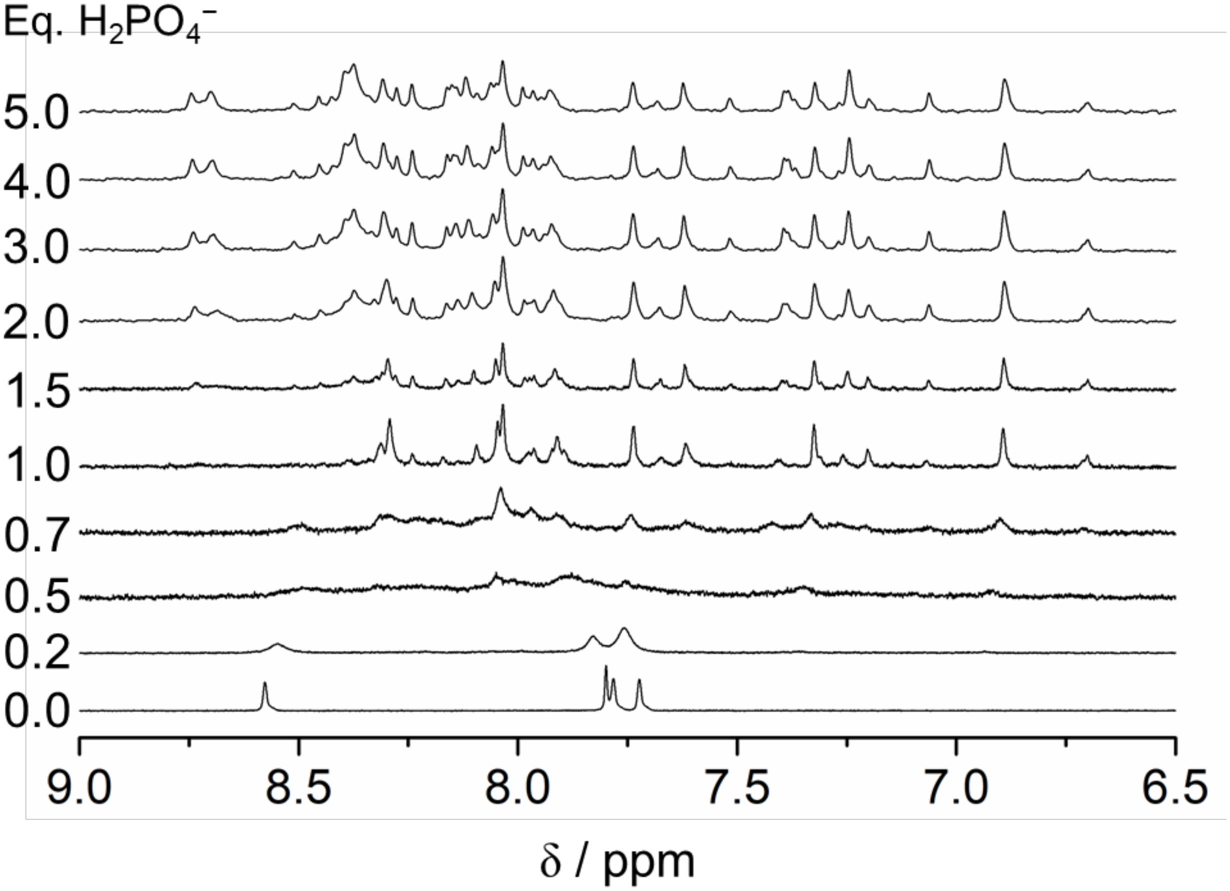

Figure S11. ^{1}H NMR titration of **CS** (1 mM) with $TBAH_2PO_4$ in 60/40 *v/v* % CD_2Cl_2/CD_3CN (600 MHz, 298 K).

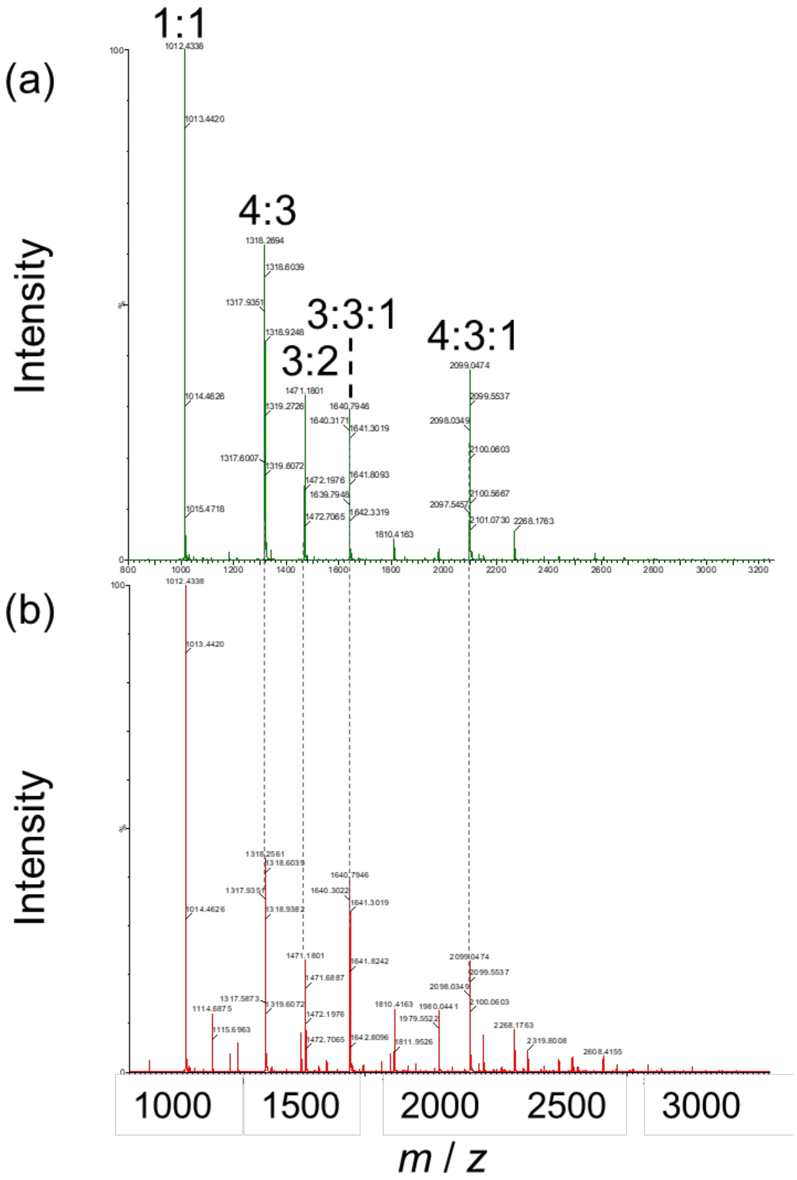

Figure S12. ESI-MS of **CS** (2 mM) with (a) 0.5 eq of $TBAH_2PO_4$ and (b) 1 eq. $TBAH_2PO_4$ in 60/40 *v*/*v* % CH_2Cl_2/CH_3CN (50 °C, 20 V, negative mode).

S7. Assignment of Stoichiometry in the 4:6:1:6 Crystal

In the crystals grown with the extra cations, there is ambiguity in the exact number of phosphates and phosphoric acids. The crystal data acquired from the crystals grown with either salicylate or BArF anions could be modelled equally well with two different stoichiometries. In the asymmetric unit, we clearly see one stacked pair of cyanostars and 3.5 phosphate species. However, there could either be 2.5 or 3.0 cations per asymmetric unit raising the unit cell's stoichiometry to 4:7:5 or 4:7:6 for **CS**:phosphate:cation. Irrespective of the ambiguities, the number of phosphate species always exceeds the maximum number of cations. This situation

strongly indicates the presence of at least one phosphoric acid. Thus, the ratios for **CS**:phosphate:phosphoric acid:cation ratios can be expressed as either 4:5:2:5 or 4:6:1:6.

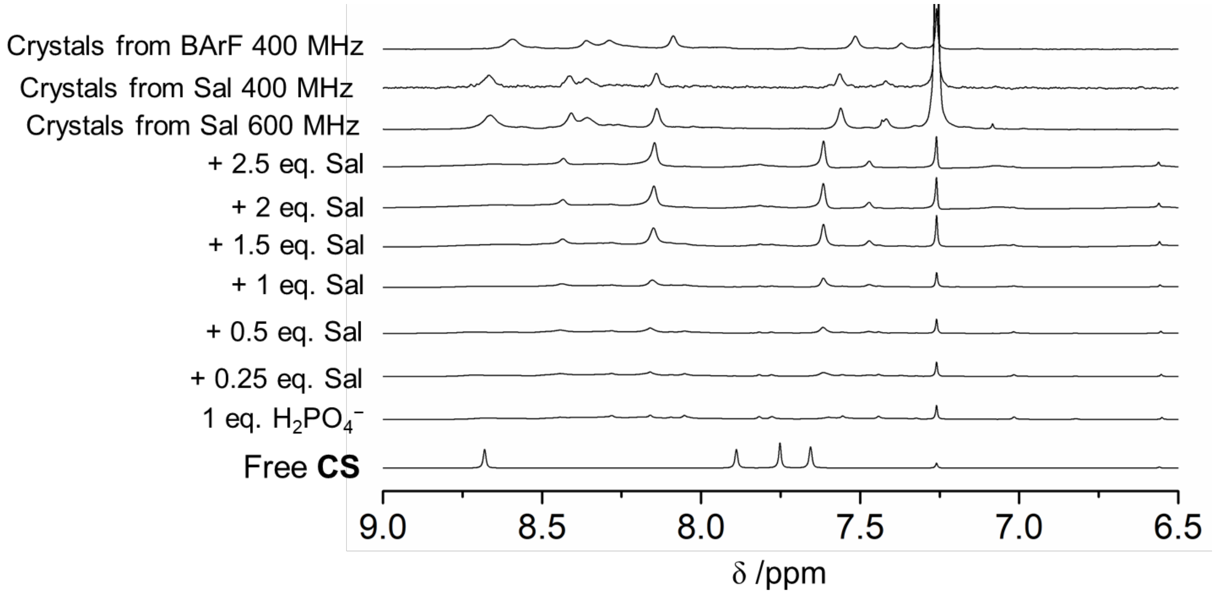

Figure S13. Comparison of NMR titrations of **CS** (10 mM) with 1 eq. $TBAH_2PO_4$ and the addition of salicylate compared to crystals obtained with **CS** grown with the co-addition of TBA(salicylate) and $TBAH_2PO_4$ and TBABArF.

As a starting point to evaluate the ratio of species using NMR spectroscopy of the dissolved crystals ($CDCl_3$), we see a ratio of intensities matching 4:*x*:6 ratio for cyanostar:phosphate species:cation. For the salicylate and BArF crystals, the errors in the intensity ratios between **CS** and NBu_4^+ are ±0.2 and ±0.1, respectively. The 4:*x*:6 ratio correlates best with the phosphate species (*x*) being equivalent to six phosphates and one phosphoric acid per unit cell. This ratio (4:6:1:6) reduces to 1.5 eq. of the salt relative to 1 eq. of cyanostar. Consistently, the dissolved crystal matches the titration of cyanostar ($CDCl_3$) with 1-1.5 eq of added phosphate (Figure S13). Crystals grown using the BArF salt showed the same behavior. The ^{31}P NMR position of the 4:6:1:6 NBu_4BArF sample (Figure S2) shows only one relatively sharp resonance at 2 ppm. This ^{31}P NMR matches that seen with 2 eq. of $NBu_4H_2PO_4$ added to cyanostar in $CDCl_3$, which is assigned to a 2:2 complex in fast exchange with excess phosphate in solution. The totality of the NMR data are more consistent with the 4:6:1:6 ratio but the other option cannot be totally excluded.

For the crystals grown with either salt, the phosphate's protons were placed with equivalent partial site occupancy on all oxygen atoms such that the overall occupancy equates to three phosphate anions and 0.5 phosphoric acids per asymmetric unit. There was some ambiguity in whether there were 2.5 or 3 tetrabutylammonium cations per asymmetric unit, but this ambiguity did not extend to the absence of a neutral species present, i.e., a valid solution would never have an equal match of cation to anion stoichiometry because there is no void large enough to accommodate another 0.5 TBA cation in the asymmetric unit.

Table S1: Comparison of crystal parameters

Parameter	Crystal structure [**CS**$_4$(H_2PO_4)$_3$(TBA)$_3$] CCDC 1588590	Crystal structure [**CS**$_4$(H_2PO_4)$_6$(H_3PO_4)(TBA)$_6$] CCDC 1588591
Formula	$C_{308}H_{374}N_{23}O_{12}P_3$	$C_{480}H_{508.52}N_{26}O_{29.28}P_7$
M / g mol^{-1}	4683.19	6457.80
Temperature / K	230(2)	173(2)
λ / Å	0.71073	0.71073
Crystal system	triclinic	triclinic
Space Group	*P*−1	*P*−1
Crystal color	colorless	colorless
Crystal size / mm	0.21×0.19×0.16 mm^3	0.31 ×0.25 ×0.24 mm^3
a	21.138(2)	19.6920(15)
b	21.616(2)	22.3708(16)
c	21.948(2)	25.201(2)
α	118.123(5)	97.908
β	106.659(6)	108.437(3)
γ	99.169(6)	113.456(3)
V/Å^3	7942.5(14)	9208.1(12)
F_{000}	2524	3466
Z	1	1
Calculated density / g cm^{-3}	0.979	1.165
Absorption Coefficient (mm)	0.074	0.200
Reflections collected	27892	37800
Reflections observed	9529	24822
Data/restraints/parameters	27892/5061/1898	37800/5557/2593
GOOF	1.343	1.021
Final R indices / %	13.99	9.00
R indices (all data) / %	43.07	30.69
Largest diff peak and hole e Å^{-3}	0.556/−0.364	1.061/−0.817

S8. X-Ray Diffraction Analyses

CS$_4$(H_2PO_4)$_3$(TBA)$_3$ (CCDC 1588590)

A colorless crystal (approximate dimensions 0.16 × 0.19 × 0.21 mm^3) was placed onto the tip of a MiTeGen loop and mounted on a Bruker APEX II Kappa Duo diffractometer equipped with an APEX II detector at 230(2) K.

Data Collection

The data collection was carried out using Mo Kα radiation (graphite monochromator) with a frame time of 180 seconds and a detector distance of 5.00 cm. A collection strategy was calculated and complete data to a resolution of 0.70 Å with a redundancy of 4 were collected. Four major sections of frames were collected with 0.50º ϕ and ω scans. Data were only observed

to about 1.2 Å, however, data to a resolution of 0.84 Å had to be considered in the reduction. The total exposure time was 52.25 hours. The frames were integrated with the Bruker SAINT software[4] package using a narrow-frame algorithm. The integration of the data using a triclinic unit cell yielded a total of 49396 reflections to a maximum θ angle of 25.17° (0.84 Å resolution), of which 27892 were independent (average redundancy 1.771, completeness = 97.8%, R_{int} = 5.25%, R_{sig} = 11.84%) and 9529 (34.16%) were greater than 2σ(F2). The final cell constants of a = 21.138(2) Å, b = 21.616(2) Å, c = 21.948(2) Å, α = 118.123(5)°, β = 106.659(6)°, γ = 99.169(6)°, volume = 7942.0(14) $Å^3$, are based upon the refinement of the XYZ-centroids of 5626 reflections above 20 σ(I) with 4.559° < 2θ < 41.52°. Data were corrected for absorption effects using the multi-scan method (SADABS[5]). The ratio of minimum to maximum apparent transmission was 0.806. The calculated minimum and maximum transmission coefficients (based on crystal size) are 0.6004 and 0.7452.

Structure solution and refinement

The space group P-1 was determined based on intensity statistics and the lack of systematic absences. The structure was solved and refined using the SHELX suite of programs.[6] An intrinsic-methods solution was calculated, which provided most non-hydrogen atoms from the E-map. Full-matrix least squares / difference Fourier cycles were performed, which located the remaining non-hydrogen atoms. All non-hydrogen atoms were refined with anisotropic displacement parameters. The hydrogen atoms were placed in ideal positions and refined as riding atoms with relative isotropic displacement parameters. Residual electron density was found near 0 0.5 0 and 0 0.5 0.5 with peak electron densities very low, yet patterned like $CHCl_3$. Refinement of crystalized solvent with partial occupancy did not yield a sensible model. Therefore, the structure was investigated for solvent accessible areas.[7] Voids were found at the aforementioned positions, which are crystallographic inversion centers, with 1081 $Å^3$ per unit cell and containing 293 electrons per unit cell. For comparison, chloroform occupies ca. 98 $Å^3$ with 58 electrons.[8] The contribution of the unidentified solvent to the structure factors was assessed by back-Fourier transformation and the data were corrected accordingly. The refinement using the modified dataset improved the overall structure and R1 by about 7 %. The final anisotropic full-matrix least-squares refinement on F2 with 1898 variables converged at R1 = 13.99%, for the observed data and wR2 = 43.07% for all data. The goodness-of-fit was 1.343. The largest peak in the final difference electron density synthesis was 0.556 $e^-/Å^3$ and the largest hole was –0.364 $e^-/Å^3$ with an RMS deviation of 0.068 $e^-/Å^3$. On the basis of the final model, the calculated density was 0.979 g/cm^3 and F(000), 2524 e^-.

CS$_2(H_2PO_4)_3(H_3PO_4)_{0.5}(TBA)_3$ (CCDC 1588591)

A colorless crystal (approximate dimensions 0.31 × 0.25 × 0.24 mm^3) was placed onto the tip of a MiTeGen loopand mounted on a Bruker APEX II Kappa Duo diffractometer equipped with an APEX II detector at 173(2) K.

Data Collection

The data collection was carried out using Mo Kα radiation (graphite monochromator) with a frame time of 120 seconds and a detector distance of 40 mm. A collection strategy was calculated and complete data to a resolution of 0.70 Å with a redundancy of 4 were collected. Four major sections of frames were collected with 0.50º ω and φ scans; details are below.

**Axis**	**2θ/°**	**ω/°**	**φ/°**	**χ/°**	**Frames**
Phi	14.50	-31.53	-346.66	30.74	739
Phi	-23.00	-13.54	-49.24	-84.11	739
Omega	-5.50	-37.75	-11.96	80.74	68
Omega	-13.00	-54.84	-258.16	64.29	91

A total of 1637 frames were collected. The total exposure time was 54.57 hours. The frames were integrated with the Bruker SAINT software package using a narrow-frame algorithm. The integration of the data using a triclinic unit cell yielded a total of 136054 reflections to a maximum θ angle of 26.47° (0.80 Å resolution), of which 37800 were independent (average redundancy 3.599, completeness = 99.4%, R_{int} = 3.73%, R_{sig} = 3.87%) and 24822 (65.67%) were greater than 2σ(F2). The final cell constants of a = 19.6920(15) Å, b = 22.3708(16) Å, c = 25.201(2) Å, α = 97.908(4)°, β = 108.437(3)°, γ = 113.456(3)°, volume = 9208.2(12) Å3, are based upon the refinement of the XYZ-centroids of 9393 reflections above 20 σ(I) with 4.595° < 2θ < 52.14°. Data were corrected for absorption effects using the multi-scan method (SADABS[5]). The ratio of minimum to maximum apparent transmission was 0.877. The calculated minimum and maximum transmission coefficients (based on crystal size) are 0.6538 and 0.7454.

Structure solution and refinement

The space group P-1 was determined based on intensity statistics and the lack of systematic absences. The structure was solved and refined using the SHELX suite of programs.[6] An intrinsic-methods solution was calculated, which provided most non-hydrogen atoms from the E-map. Full-matrix least squares / difference Fourier cycles were performed, which located the remaining non-hydrogen atoms. All non-hydrogen atoms were refined with anisotropic displacement parameters with exception of those of one chloroform and ether molecule disordered with one another, which remained isotropic. The hydrogen atoms were placed in ideal positions and refined as riding atoms with relative isotropic displacement parameters. Cations, anions, solvents and cyanostar molecules are disordered and were refined with restraints and constraints. In particular the phosphate distances have been restrained and averaged because of disorder and should not be interpreted. Hydrogen atoms on the phosphate were placed and refined using a riding model, such that per asymmetric unit on all phosphate oxygen atoms a partially occupied and riding hydrogen atom was placed to sum to one half neutral H_3PO_4 and three $H_2PO_4^-$ to balance the charge of three TBA cations. This choice was made because of the disorder of the anions but would be consistent with a model that hydrogen atoms are rather mobile along the hydrogen-bonded phosphate chain. The final anisotropic full-matrix least-squares refinement on F2 with 2593 variables converged at R1 = 9.00%, for the observed data

and wR_2 = 30.69% for all data. The goodness-of-fit was 1.021. The largest peak in the final difference electron density synthesis was 1.061 $e^{-}/Å^3$ and the largest hole was –0.817 $e^{-}/Å^3$ with an RMS deviation of 0.077 $e^{-}/Å^3$. On the basis of the final model, the calculated density was 1.165 g/cm^3 and F(000), 3466 e^{-}. The final model contains solvent accessible voids totaling 234 $Å^3$ to contain 53 e^{-} per unit cell with the larges void of 205$Å^3$ containing 53 e^{-} at the crystallographic inversion center at 0 0 ½.[7] For reference a TBA cation occupies 424.4 Å with 139 e^{-}.[8]

References

1. S. Lee, C.-H. Chen and A. H. Flood, *Nat. Chem.*, 2013, **5**, 704-710.
2. E. M. Fatila, E. B. Twum, A. Sengupta, M. Pink, J. A. Karty, K. Raghavachari and A. H. Flood, *Angew. Chem. Int. Ed.*, 2016, **55**, 14057-14062.
3. E. M. Fatila, E. B. Twum, J. A. Karty and A. H. Flood, *Chem. Eur. J.*, 2017, **23**, 10652-10662.
4. Bruker, *Bruker Analytical X-ray Systems*, 2016.
5. R. Blessing, *Acta Cryst. A*, 1995, **51**, 33-38.
6. G. Sheldrick, *Acta Cryst. A*, 2008, **64**, 112-122.
7. A. Spek, *Acta Cryst. C*, 2015, **71**, 9-18.
8. A. Bondi, *J. Phys. Chem.*, 1964, **68**, 441-451.
